# Efficacy of a nurse-led sexual rehabilitation intervention for women with gynaecological cancers receiving radiotherapy: results of a randomised trial

**DOI:** 10.1038/s41416-024-02775-8

**Published:** 2024-07-03

**Authors:** Isabelle Suvaal, Susanna B. Hummel, Jan-Willem M. Mens, Charlotte C. Tuijnman-Raasveld, Roula Tsonaka, Laura A. Velema, Henrike Westerveld, Jeltsje S. Cnossen, An Snyers, Ina M. Jürgenliemk-Schulz, Ludy C. H. W. Lutgens, Jannet C. Beukema, Marie A. D. Haverkort, Marlies E. Nowee, Remi A. Nout, Cor D. de Kroon, Wilbert B. van den Hout, Carien L. Creutzberg, Helena C. van Doorn, Moniek M. ter Kuile

**Affiliations:** 1 Department of Obstetrics and Gynaecology, Leiden University Medical Center, Leiden, The Netherlands; 2 Department of Radiotherapy, Erasmus MC Cancer Institute, University Medical Center Rotterdam, Rotterdam, The Netherlands; 3 Department of Biomedical Data Sciences, Leiden University Medical Center, Leiden, The Netherlands; 4 Department of Radiation Oncology, Leiden University Medical Center, Leiden, The Netherlands; 5 Department of Radiation Oncology, Amsterdam UMC Location University of Amsterdam, Amsterdam, The Netherlands; 6 Department of Radiation Oncology, Catharina Hospital, Eindhoven, The Netherlands; 7 Department of Radiation Oncology, Radboudumc, Nijmegen, The Netherlands; 8 Department of Radiation Oncology, University Medical Center Utrecht, Utrecht, The Netherlands; 9 Department of Radiation Oncology, Maastro, Maastricht, The Netherlands; 10 Department of Radiation Oncology, University Medical Center Groningen, Groningen, The Netherlands; 11 Department of Radiation Oncology, Radiotherapiegroep, Arnhem, The Netherlands; 12 Department of Radiation Oncology, The Netherlands Cancer Institute, Amsterdam, The Netherlands; 13 Department of Gynaecology, Erasmus MC, University Medical Center Rotterdam, Rotterdam, The Netherlands

**Keywords:** Gynaecological cancer, Human behaviour

## Abstract

**Background:**

The multicentre randomised SPARC trial evaluated the efficacy of a nurse-led sexual rehabilitation intervention on sexual functioning, distress, dilator use, and vaginal symptoms after radiotherapy for gynaecological cancers.

**Methods:**

Eligible women were randomised to the rehabilitation intervention or care-as-usual. Four intervention sessions were scheduled over 12 months, with concurrent validated questionnaires and clinical assessments. Primary outcome was the Female Sexual Function Index (FSFI). A generalised-mixed-effects model compared groups over time.

**Results:**

In total, 229 women were included (*n* = 112 intervention; *n* = 117 care-as-usual). No differences in FSFI total scores were found between groups at any timepoint (*P* = 0.37), with 12-month scores of 22.57 (intervention) versus 21.76 (care-as-usual). The intervention did not significantly improve dilator use, reduce sexual distress or vaginal symptoms compared to care-as-usual. At 12 months, both groups had minimal physician-reported vaginal stenosis; 70% of women were sexually active and reported no or mild vaginal symptoms. After radiotherapy and brachytherapy, 85% (intervention) versus 75% (care-as-usual) of participants reported dilation twice weekly.

**Discussion:**

Sexual rehabilitation for women treated with combined (chemo)radiotherapy and brachytherapy improved before and during the SPARC trial, which likely contributed to comparable study groups. Best practice involves a sexual rehabilitation appointment 1 month post-radiotherapy, including patient information, with dilator guidance, preferably by a trained nurse, and follow-up during the first year after treatment.

**Clinical trial registration:**

NCT03611517.

## Background

Women with locally advanced cervical and vaginal cancer are primarily treated with external beam radiotherapy with concurrent cisplatin-based chemotherapy and MRI-guided adaptive brachytherapy. Those with early-stage cervical or endometrial cancer treated with upfront surgery receive adjuvant external beam radiotherapy (with or without brachytherapy boost) in case of lymph node involvement, close or involved surgical margins or a combination of risk factors. The impact of these gynaecological cancer treatments on sexual functioning can be substantial and is more pronounced when radiotherapy is included, as compared to surgery alone [1, 2]. Especially treatment with both external beam radiotherapy and brachytherapy has been shown to impact vaginal and sexual functioning by causing morphological changes in the vaginal mucosa, such as atrophy, adhesions, and fibrosis which may lead to vaginal stenosis and shortening [3–5].

Regular vaginal dilation has been shown to help prevent and reduce vaginal stenosis [6]. However, many women (75%) fail to use dilators regularly, even with counselling and specific instructions [7, 8]. Some studies suggested that additional professional support, including psycho-education and motivation, can improve compliance [6], but not all studies showed such benefit [9, 10]. In addition, reported interventions targeting dilator use did not address other psychosexual consequences of treatment of gynaecological cancer, such as sexual distress and (worries about) pain during intercourse [3, 7, 11].

Some small studies have investigated psychosexual interventions such as cognitive behavioural techniques, psycho-education and counselling to address sexual problems after radiotherapy [5, 11–13]. Results indicated that such interventions can lead to improved sexual functioning, reduced sexual distress, and when the partner was actively involved, enhanced relationship satisfaction.

In a previous pilot study, a specifically developed nurse-led sexual rehabilitation intervention combining psycho-education and cognitive behavioural therapy was shown to improve sexual functioning and compliance with dilator use in women treated with chemoradiotherapy and brachytherapy [14]. Subsequently a randomised trial was designed to evaluate the effectiveness of this rehabilitation intervention as compared to care-as-usual. We hypothesised that women receiving this nurse-led rehabilitation intervention would experience significantly greater improvement in sexual functioning at 12 months after radiotherapy. In addition, we anticipated improved compliance with dilator use and fewer vaginal functioning problems and sexual distress.

## Methods

### Study design and participants

The SPARC (Sexual rehabilitation Programme After Radiotherapy for gynaecological Cancer, NCT03611517) study was a multicentre randomised trial conducted in all 10 Dutch gynaecological oncology centres. Participating centres, including their study teams, are listed in Supplementary Appendix 1 (p. 1). A detailed description of the trial design has been previously reported [15].

Before start of the trial, a study-specific 50-h training programme was held, to which each participating centre sent at least two designated oncology nurses (for details, see Table 1) [14, 15]. Only after completing this programme, nurses were allowed to conduct the intervention. An additional training programme and annual focused training days were organised during the years of the study.

**Table 1:** Description of the sexual rehabilitation programme.

**Fig. 1:**
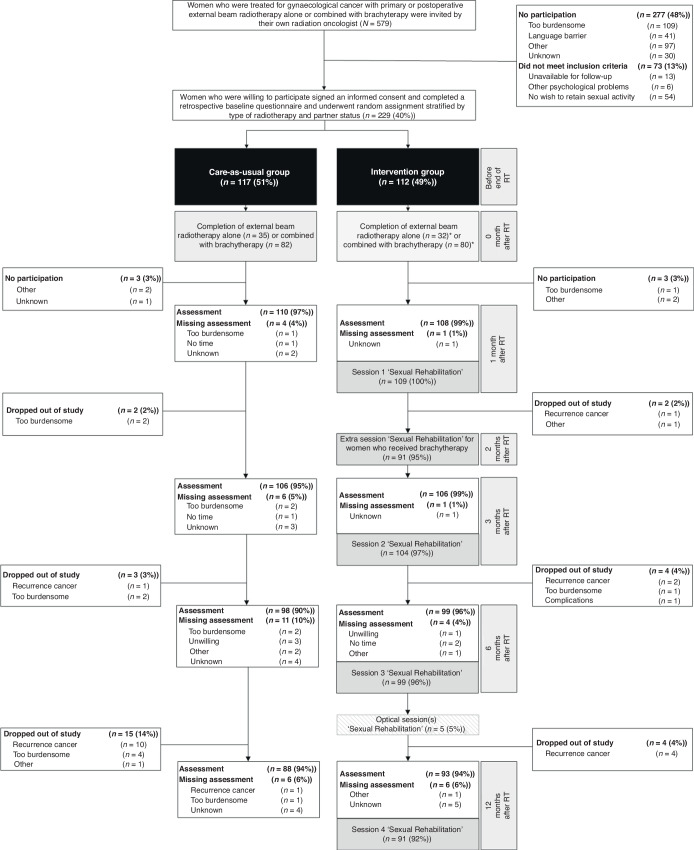
CONSORT diagram. *N* = total number of patients invited for the study, *n* = subgroup of patients, RT = radiotherapy. *One patient was initially stratified for external beam radiotherapy combined with brachytherapy but ultimately received external beam radiotherapy alone. Consequently, her rehabilitation trajectory was according to external beam radiotherapy alone; therefore she was moved to external beam radiotherapy alone. ^x^One patient was initially stratified for external beam radiotherapy alone, however she received an additional external beam radiotherapy boost. This led to modification in her rehabilitation trajectory to align external beam radiotherapy combined with brachytherapy; therefore she was moved to external beam radiotherapy combined with brachytherapy.

General features
*Nurses*
Each participating centre sent at least two designated oncology nurses; or brachytherapy technicians (two centres); or radiotherapy medical assistants (one centre). In total, 25 oncology nurses were involved in the study, divided over 10 Dutch oncology centres.
The training programme was developed during the pilot study by one clinical psychologist, and two healthcare psychologists, all with at least 25 years expertise in sexology, conceptualisation, methods and skills [14].
Prior to the start of the trial, all nurses completed a 50-h study-specific training in the basic principles of sexology, motivational interviewing, simple cognitive behavioural interventions, and the treatment protocol itself. Because of changes in employment, the training was repeated in 2020 for new nurses from some participating centres and for an additional participating centre.
The nurses were supervised by experienced sexologists (*N* = 12), who were also trained in the treatment protocol.
Over a period of ~4 years, the nurses attended six additional training days that focused on a specific theme that was relevant for the study (i.e., vaginal stenosis and dilator use; emotional reactions after loss of participants due to cancer recurrence; the partner relationship; implementation of the intervention). During five of these training days, the supervisors were also present. One of these training days took place online due to the COVID-19 pandemic.
*Structure of the sexual rehabilitation programme (see also Fig.* *1**)*
The intervention consisted of four 1-h face-to-face sessions at 1, 3, 6 and 12 months after radiotherapy.
An additional session was scheduled at 2 months after radiotherapy for women who received external beam radiotherapy combined with brachytherapy, during which potential barriers and problems with dilator use were discussed.
An extra follow-up session/telephone consultation of 30 min was scheduled between 6 and 12 months post-treatment, if preferred by the participant.
The sexual rehabilitation intervention consisted of 11 modules (see also description of modules below). The modules included topics such as education regarding the specific cancer diagnosis, treatment and importance of long-term regular dilator use, discussing potential experienced barriers to dilator or lubricant use, fear of penetration with dilators and resuming sexual activity, promoting couples’ mutual coping and support processes and addressing sexual, body image and relationship concerns. If sexual problems appeared to beyond the scope of the modules, referral options to a psychologist-sexologist were given.
The content of the intervention was personalised for each individual person and was tailored to the participant-specific psychological, relational and somatic factors. During each session, the nurse selected the specific module(s) that fitted the woman’s (and her partner’s) needs best. See Suvaal et al. [15] for the decision tree for module selection.
The sessions were designed to be face-to-face; however, during the COVID-19 pandemic, sessions could also take place by telephone or video.
*Involvement of the partner*
Partners were invited and encouraged to accompany the sessions. Their presence was, however, not obligatory.
*Costs of the sexual rehabilitation programme*
The sexual rehabilitation programme was provided at no cost to the women.
*Description of modules*
Module 1: Brief sexual history
This module describes how the nurse can question the patient in-depth about sexual problems on various domains of sexual functioning, including sexual interest/ arousal, orgasm, pain and sexual satisfaction. It also covers psycho-education about sexuality and the sexual response curve and provides information about frequently occurring sexual problems and solutions.
Module 2: Pain during intercourse
This module includes practical guidelines that the nurse can provide regarding pain during intercourse after radiotherapy-, with referrals to modules 3, 4, 6 and 7, and explains how to provide psycho-education about the circular model of dyspareunia, which is based on a cognitive behavioural framework.
Module 3: Vaginal dryness and health
This module provides the nurse with instructions on how to give advice with regard to treatment of vaginal dryness, pain or irritation. It also includes information regarding vaginal health, such as the use of vaginal creams, avoidance of scratching in response to irritated skin or avoidance of washing with soap.
Module 4: Alternatives for intercourse
The exercise in this module helps the woman and her partner (if available) to explore and discuss non-penetrative alternatives for sexual intercourse.
Module 5: The partner and possible sexual problems
This module can be consulted by the nurse when partners experience temporary sexual problems, such as erectile dysfunction during intercourse. The module also includes a reference to module 1.
Module 6: Gradual exposure towards sexual intercourse
The aim of the steps in this module, which are based on a cognitive behavioural gradual exposure therapy for Genito-Pelvic/Penetration Disorder, is to learn about the woman and her partner how to re-engage in sexual intercourse. The steps include: touching of the vaginal opening with the erect penis without penetration, stepwise vaginal insertion of the erect penis without moving, and vaginal insertion of the erect penis with moving.
Module 7: Pelvic floor exercise
This module includes several pelvic floor relaxation exercises for women who experience tension in the pelvic floor muscles.
Module 8: Difficulties with dilator use at home
This module is suitable for women who experience problems with dilator use and who already practiced under supervision of a nurse (see module 9) or for women who do not want to practice under supervision. This module provides the nurse with instructions on how to give specific advice on how to overcome experienced difficulties, after first exploring the problems during dilator use (e.g. pain/discharge, loss of blood or difficulties with inserting the dilator).
Module 9: Using dilators under supervision at the outpatient clinic
This module focuses on women who experience fear with regard to dilator use or who experience difficulties when using vaginal dilators, due to for example tension of the pelvic floor. The nurse-led session is based on therapist-aided exposure therapy for Genito-Pelvic/Penetration Disorder. The goal is to reduce fear of dilator use by using a stepwise exposure session in which the woman—who performs the vaginal dilation by herself—is facilitated by the nurse. During the session, tips are given with regard to a correct and more comfortable use of the dilators. Furthermore, the nurse helps to evaluate and articulate any unhelpful cognitions about what could (or could not) occur during dilator use. In these instances, the exposure is used as a behavioural experiment, to test the tenability of these cognitions. The module also includes advice on how to handle problems that might occur during practicing at home.
Module 10: Exploring and resolving ambivalence with regard to dilator use
The aim of the exercise in this module is to motivate the woman for dilator use, by acknowledging, exploring and resolving ambivalent feelings towards dilator use by motivational interviewing technique. By exploring pros and cons of both dilator use and no dilator use, the woman can be supported in making an informed choice about dilator use. If she decides to use dilators, problems with dilator use are discussed in more detail and how to overcome them. If a woman decides not to use dilators, tampons covered in petroleum jelly (Vaseline) are recommended and guidelines on how to use these are provided to the woman (see module 11).
Module 11: Petroleum jelly (Vaseline) tampons
This module follows module 10, when a woman decides not to use dilators. The module covers guidelines on how to use tampons covered in petroleum jelly (Vaseline).

*N* = total number of supervisors involved in the study.

*h* hour, *min* minutes.

Eligible women had a histological diagnosis of cervical, vaginal or endometrial cancer; received primary or postoperative external beam radiotherapy with or without concurrent chemotherapy and brachytherapy, or postoperative radiotherapy alone; were 18 years or older, and intended to retain sexual activity. Both single and partnered women, regardless of their sexual orientation, could participate.

Exclusion criteria were unavailability for follow-up; insufficient Dutch language proficiency; major affective, psychotic or substance abuse disorder, or posttraumatic stress disorder related to pelvic floor/genital abuse.

The radiation oncologists at the participating centres screened potential participants. Eligible women were informed about the background, rationale and specifics of the study protocol. All participating women provided written informed consent and completed a baseline questionnaire before completion of radiotherapy. In this baseline questionnaire they retrospectively completed questions about sexual functioning and distress prior to cancer symptoms and diagnosis.

The protocol was approved by the Scientific Review Board of the Dutch Cancer Society, by the Medical Ethics Committee Leiden-Den Haag-Delft (number NL62767.058.17), and by the Institutional Review Boards and/or Ethics Committees of the participating centres.

### Randomisation and masking

Participants were assigned unique study identifiers by the local data manager for use in all questionnaires and data files. Participants were randomly assigned (1:1) to the nurse-led sexual rehabilitation intervention or care-as-usual, using block stratified randomisation (block sizes of 2 and 4). Stratification was based on radiotherapy type (brachytherapy yes/no) and partner status (yes/no). Participants were registered by the local data manager through a secured web-based system, and randomised after completing baseline measurements. Participants, physicians, nurses, and investigators were not masked to treatment allocation.

### Procedures

In the intervention group, all women were counselled and followed by a specifically trained nurse [14]. The content of this nurse-led sexual rehabilitation intervention has been described in detail elsewhere [15] and is summarised in Table 1 and Fig. 1. In short, the intervention comprised four 1-h face-to-face sessions at 1, 3, 6 and 12 months post-radiotherapy, synchronised with visits to the radiation oncologist, with an extra session at 2 months for women who received brachytherapy. All nurse-led intervention sessions were audio-taped for checks of adherence to the protocol and assessment of competence by an independent panel. The aim was to conduct random checks of 15% of the sessions, which is customary in this type of research, where a minimum of 10% is considered acceptable within large cohorts [16].

Both the intervention and care-as-usual groups had a first follow-up session 4–5 weeks after completion of radiotherapy with their radiation oncologist, to evaluate recovery, tumour regression and vaginal healing, and to assess symptoms. All women received a specially developed information booklet which was based on the pilot study [14]. Those who had received radiotherapy with brachytherapy also received a vaginal dilator set (Amielle Comfort®; Owen Mumford) and two tubes of lubrication gel (K-Y Jelly; Johnson & Johnsen) free of charge. They were advised to start vaginal dilation for 1–3 min, 2–3 times a week, provided the vagina was sufficiently healed, and to continue regular vaginal dilation throughout the first year after radiotherapy. If sexual intercourse was resumed, this was also considered as part of vaginal dilation, which could be complemented with the use of the dilator set. Women with cervical or vaginal cancers who were under 50 years of age were recommended to receive hormone replacement therapy until the age of about 50.

Prior to the study, the study team at the centres had been queried about their standard protocols regarding sexual rehabilitation within their centre (‘care-as-usual’). In most of the centres (90%), specific counselling on sexual rehabilitation and dilation was already a standard topic of information after treatment and during follow-up appointments with their physician. Although the guidance offered to the care-as-usual group could not be completely standardised due to these local practices, it did not involve the structured, tailored nurse-led sexual rehabilitation intervention during follow-up.

### Outcomes

The primary outcome was overall sexual functioning, as measured with the Female Sexual Function Index (FSFI) [17]. A total score of ≤26.55 has been validated as the cut-off score for diagnosis of female sexual dysfunction [18]. We added two questions to the FSFI to assess the frequency of sexual activity with and without sexual intercourse. Secondary outcomes included sexual distress, as measured by the Female Sexual Distress Scale (FSDS; scores ≥15 have been signified to establish the presence of sexual distress) [19], compliance with dilator use (assessed using a 4-item questionnaire regarding frequency, duration, sexual intercourse and other vaginal penetration activities), vaginal functioning problems such as shortness, dryness and pain during intercourse (measured by the Cervical Cancer Module of the European Organization for Research and Treatment of Cancer (QLQ-CX24) [20]), and physician-reported vaginal dryness, shortening and/or tightening, and dyspareunia (assessed by standardised clinical examination using the Common Terminology Criteria for Adverse Events (CTCAE, version 4.03)). See Supplementary Appendix 2 (p. 2) for additional specific patient and physician-reported outcome measures, with cut-off scores if applicable.

Outcomes were assessed before radiotherapy (retrospective baseline) and 1, 3, 6 and 12 months after radiotherapy. To minimise respondent burden, the baseline questionnaire included only the FSFI and the FSDS. Adverse events related to dilator use were documented. Cancer treatment-related adverse events were not considered study-related.

### Statistical analysis

An effect size of *d* = 0.50 indicates a moderate and clinically relevant effect size [21]. This corresponds with a difference of 3.4 points on the primary outcome measure (FSFI), with a standard deviation of 6.8. To achieve 80% power at a 0.05 significance level, each study group required a minimum of 64 evaluable women at 12 months. Considering the 40% dropout rate observed in the pilot study [14], at least 107 women in both the intervention and care-as-usual group were required, stratified by radiotherapy type and partner status.

During participant recruitment, it became evident that women undergoing both external beam radiotherapy and brachytherapy were more likely to participate, probably due to more advanced age and milder side effects in those receiving pelvic radiotherapy alone. Consequently, the study population comprised relatively young women with cervical carcinoma primarily treated with external beam radiotherapy, concurrent cisplatin-based chemotherapy, and image-guided adaptive brachytherapy. Given the intervention’s relevance to these younger women with intensive treatment for cervical and vaginal carcinomas, we decided to continue the enrolment of eligible women regardless of radiotherapy type to a total of 220 women. The study was amended accordingly.

Analyses were based on intention-to-treat and were conducted with the Statistical Package for Social scientists (version 29) and the GLMM-adaptive package in R (version 4.2.1) [22]. Questionnaire scores were calculated using published algorithms [17, 19, 20]. Missing values were replaced by the average score of completed items on the same scale for each individual, when ≥75% of items were completed. When a scale consisted of only two items, 100% of the items had to be completed.

To address differences in changes in the primary and secondary outcome measures between groups (intervention versus care-as-usual) over time we modelled either the mean scores, log expected counts or log odds (depending on the type of variable) as a function of time, of the intervention group and their interaction. In addition, radiotherapy with or without brachytherapy was added as factor to the model. For the physician-reported variables, when fewer than 15 women scored within CTCAE grade 2 and/or 3 events, these scores were combined into a single category with CTCAE grade 1 (‘toxicity’), and then compared to CTCAE grade 0 (‘no toxicity’). Differences between groups were evaluated based on a generalised linear mixed effects model, specifically depending on the outcome considered we used mixed effects poisson regression (count measurements) or mixed effects logistic regression (dichotomous outcomes). We used a beta distribution instead of a normal distribution for our continuous outcomes, because of the bounded nature of these outcomes (e.g. the FSFI total score takes values between 2 and 36 [17]). In addition, we used a random effects model (i.e., random intercepts and random slopes) to capture the within-subjects correlation. We further explored if there was considerable between-hospital variability. Regarding missing data, the generalised linear mixed effects models give valid results under the missing at random mechanism. To account for any potential model misspecification robust standard errors were computed. We used the Likelihood Ratio Test to test whether the improvement of the intervention group over time was statistically significantly different from the care-as-usual group. The significance level was set at 0.05.

This study was monitored for trial and data compliance by an independent certified monitor and registered under ClinicalTrials.gov number NCT03611517.

## Results

Between Aug 7, 2018, and Dec 31, 2021, 229 women were enrolled and randomly assigned to the nurse-led sexual rehabilitation intervention (*n* = 112, 49%) or to care-as-usual (*n* = 117, 51%) (see Fig. 1). 36 women (16%) discontinued participation before the 12-month assessment. Dropout was significantly lower in the intervention group (*n* = 13, 12%) than in the care-as-usual group (*n* = 23, 20%) (*P* < 0.001). Follow-up and/or questionnaire data of 39 women (17%) were not available at one or more timepoints during the 12-month follow-up period: 12 (11%) in the intervention group; 27 (23%) in the care-as-usual group).

Patient, disease and treatment characteristics were well-balanced between the two groups (see Table 2). Most women were treated with primary or postoperative external beam radiotherapy combined with brachytherapy (80 (71%) in the intervention group; 82 (70%) in the care-as-usual group) for cervical cancer (98 (87.5%) for the intervention group; 104 (89%) for the care-as-usual group), and had a partner (88 (79%) for the intervention group; 90 (77%) for the care-as-usual group); with mostly male partners (86 (98%) for the intervention group; 90 (100%) for the care-as usual group). Approximately 70% of the women under the age of 50 with cervical cancer received hormonal replacement therapy during follow-up.

**Table 2:** Patient, disease and treatment characteristics.

		Intervention group, *n* = 112 (48.9%)	Care-as-usual group, *n* = 117 (51.1%)	
**Patient characteristics**				
Age	Median in years (± IQR)	42 (17)	41 (19)	
Partner	Yes	88 (78.6%)	90 (76.9%)	
	- Male	86 (97.7%)	90 (100%)	
	- Female	2 (2.3%)	0	
	No	24 (21.4%)	27 (23.1%)	
Highest completed level of education	Primary education	7 (6.3%)	6 (5.1%)	
	Secondary education	50 (44.6%)	54 (46.2%)	
	Higher education	54 (48.2%)	56 (47.9%)	
	Missing	1 (0.9%)	1 (0.9%)	
Menopausal status before diagnosis	Premenopausal	75 (67.0%)	75 (64.1%)	
	Perimenopausal	10 (8.9%)	3 (2.6%)	
	Postmenopausal	23 (20.5%)	33 (28.2%)	
	Unknown	4 (3.6%)	6 (5.1%)	
Vaginal delivery	Yes	68 (60.7%)	69 (59.0%)	
	No	40 (35.7%)	43 (36.8%)	
	Unknown	2 (1.8%)	5 (4.3%)	
	Missing	2 (1.8%)	0	
Body Mass Index	Underweight (< 18.5)	5 (4.5%)	5 (4.3%)	
	Healthy weight (18.8–24.9)	59 (52.7%)	44 (37.6%)	
	Overweight (25–19.9)	20 (17.9%)	40 (34.2%)	
	Obese (> 30)	27 (24.1%)	25 (21.4%)	
	Missing	1 (0.9%)	3 (2.6%)	
Current smoker	Yes	18 (16.1%)	14 (12.0%)	
	No	94 (83.9%)	102 (87.2%)	
	Unknown	0	1 (0.9%)	
World Health Organization performance score	0	85 (75.9%)	89 (76.1%)	
	1	24 (21.4%)	25 (21.4%)	
	2	2 (1.8%)	3 (2.6%)	
	Unknown	1 (0.9%)	0	
**Disease characteristics**				
Type of carcinoma	Cervical carcinoma	98 (87.5%)	104 (88.9%)	
	Endometrial carcinoma	7 (6.3%)	8 (6.8%)	
	Vaginal carcinoma	7 (6.3%)	5 (4.3%)	
Histological type	Cervical			
	- Squamous cell	80 (81.6%)	85 (81.7%)	
	- Other	18 (18.4%)	19 (18.3%)	
	Endometrium	5 (71.4%)	5 (62.5%)	
	- Endometrioid carcinoma	0	1 (12.5%)	
	- Serous carcinoma	2 (28.6%)	2 (25.0%)	
	- Mixed or other	6 (85.7%)	4 (80.0%)	
	Vagina	1 (14.3%)	1 (20.0%)	
	- Squamous cell			
	- Other			
FIGO stage (2009)	Cervical			
	- IB	29 (29.6%)	32 (30.8%)	
	- IIA/B	52 (53.1%)	56 (53.8%)	
	- IIIA/B	11 (11.2%)	11 (10.6%)	
	- IVA	1 (1.0%)	1 (1.0%)	
	- Not applicable (if recurrence)	5 (5.1%)	4 (3.8%)	
	Endometrium			
	- IA/B	3 (42.9%)	2 (25.0%)	
	- II	1 (14.3%)	3 (37.5%)	
	- IIIA-C	3 (42.9%)	3 (37.5%)	
	Vagina			
	- I	4 (51.7%)	2 (40.0%)	
	- II	3 (42.9%)	1 (20·0%)	
	- III	0	0	
	- IVA	0	2 (40.0%)	
Lymph node metastases	Yes	59 (52.7%)	68 (58.1%)	
	No	53 (47.3%)	49 (41.9%)	
**Treatment characteristics**				
Treatment centre	Amsterdam Medical Center	16 (14.3%)		15 (12.8%)
	Catharina hospital	13 (11.6%)		12 (10.3%)
	Erasmus Medical Center	24 (21.4%)		26 (22.2%)
	Leiden University Medical Center	23 (20.5%)		22 (18.8%)
	Maastro	6 (5.4%)		10 (8.5%)
	Netherlands Cancer Institute	3 (2.7%)		2 (1.7%)
	Radboud University Medical Center	11 (9.8%)		13 (11.1%)
	Radiotherapiegroep	2 (1.8%)		3 (2.6%)
	University Medical Center Groningen	5 (4.5%)		4 (3.4%)
	University Medical Center Utrecht	9 (8.0%)		10 (8.5%)
Chemotherapy (concurrent)	Yes	90 (80.4%)		87 (74.4%)
	No	22 (19.6%)		30 (25.6%)
Hyperthermia^a^	Yes	4 (3.8%)		15 (13.8%)
	No	101 (96.2%)		94 (86.2%)
Type of Radiotherapy	Primary EBRT + BT	80 (71.4%)		82 (70.1%)
	Postoperative EBRT + BT	16 (14.3%)		15 (12.8%)
	External Beam Radiotherapy only	14 (12.5%)^b^		19 (16.2%)
	EBRT with EBRT boost	2 (1.8%)^c^		1 (0.9%)
Target area External Beam Radiotherapy	Pelvic region	84 (75.0%)		89 (76.1%)
	Pelvic and para-aortal regions	22 (19.6%)		22 (18.8%)
	Pelvic and inguinal regions	6 (5.4%)		4 (3.4%)
	Pelvic, para-aortal, and inguinal regions	0		2 (1.7%)
External Beam Radiotherapy total dose	Median dose in Gy (± IQR)	45 (0)		45 (0)
Brachytherapy	Yes	96 (85.7%)		97 (82.9%)
	No	16 (14.3%)		20 (17.1%)
Target area Brachytherapy	Intrauterine/vaginal Brachytherapy primary	80 (83.3%)		87 (89.7%)
	Vaginal vault boost postoperative	9 (9.4%)		8 (8.2%)
	Vaginal intracavitary and interstitial (primary or recurrence)	6 (6.3%)		2 (2.1%)
	Vaginal intracavitary primary	1 (1.0)		0

*EBRT* *+* *BT* external beam radiotherapy combined with brachytherapy, *FIGO* Fédération Internationale de Gynécologie et d’Obstétrique, *IQR* interquartile range, *n* = subgroup sample.

^a^Only applicable for cervical and vaginal carcinoma.

^b^One participant was stratified as EBRT + BT radiotherapy, however she was treated with EBRT only. Her rehabilitation trajectory was according to EBRT alone; therefore she was moved to EBRT alone.

^c^One participant was stratified as EBRT alone, however, she received an additional EBRT boost. Her rehabilitation trajectory was according to EBRT + BT; therefore she was moved to EBRT + BT.

Women in the intervention group had on average 4.4 (SD = 1.1) intervention sessions, lasting on average 31 min (SD = 14.6) per session. Most sessions were face-to-face: 102 (94%), 49 (56%), 80 (77%), 68 (69%), 65 (71%) for 1, 2, 3, 6 and 12 months after radiotherapy, respectively. The participation of partners declined over time: among the 88 women in the intervention group with a partner, 40 partners (45.5%) participated at the 1-month session, 31 (35%) at 3 months, 21 (24%) at 6 months, and 14 (17%) at 12 -  months after radiotherapy. Twenty partners of the 74 partnered women in each arm in the radiotherapy with brachytherapy group (27%) participated in the 2-month session focusing on dilator use. Random checks of protocol adherence and competence assessment indicated a 90% adherence and competence rate. There were no sexual rehabilitation or dilator use-related serious adverse events.

**Fig. 2:**
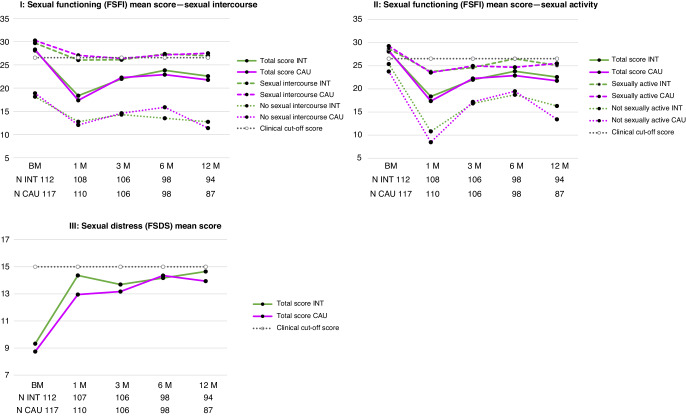
Patient-reported average scores on sexual functioning and sexual distress. BM baseline measurement, CAU Care-as-usual group, FSDS Female Sexual Distress Scale (higher score is more sexual distress), FSFI Female Sexual Function Index (higher score is better sexual functioning), INT Intervention group, M months, *N* = observed number of women at the specific timepoint.

Regarding the primary outcome FSFI, for both study groups scores were clearly decreased after radiotherapy (compared to the retrospective baseline scores), whereafter they increased over time. At 12 months, FSFI total scores were 22.57 in the intervention group versus 21.76 in the care-as-usual group, see Fig. 2I-II. As the FSFI is very sensitive to sexual activity, we also show the mean scores of sexually active women (with or without intercourse) and women not sexually active. At 12 months, 67 (71%) women in the intervention group and 60 (69%) women in the care-as-usual group were sexually active, with 65 (69%) women in the intervention group and 56 (64%) women in the care-as-usual group reporting sexual intercourse (see Supplementary Appendix 3, p. 4). As shown in Fig. 2I-II, FSFI scores for women not sexually active were lower than for sexually active women, while the pattern of decrease and increase over time was similar in both groups.

Regarding our secondary outcomes, compared to the situation prior to diagnosis, sexual distress as measured by the FSDS was strongly increased after radiotherapy, whereafter it remained elevated over time (see Fig. 2III). Almost half of the women reported sexual distress to a clinical degree at 12 months after radiotherapy in both study groups (42 (45%) women in the intervention group versus 40 (46%) women in the care-as-usual group, see Supplementary Appendix 6, p. 17).

**Fig. 3:**
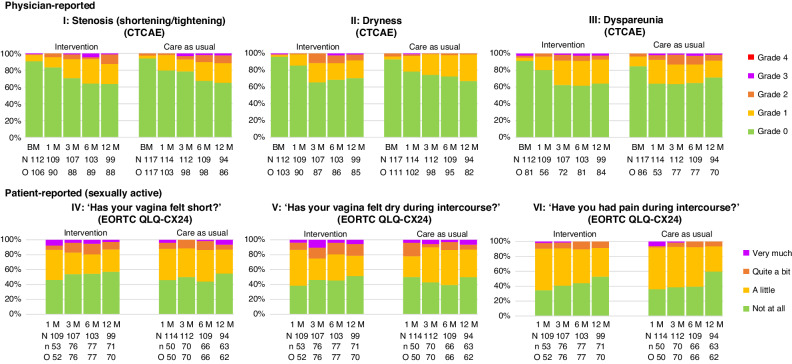
Physician-reported clinical measurements and patient-reported vaginal functioning problems (of sexually active women in the past 4 weeks) on a single-item level over time. The proportion of women is shown in percentages. BM baseline measurement, CTCAE Common Terminology Criteria for Adverse Events, EORTC QLQ-CX24 European Organization for Research and Treatment of Cancer Quality of Life Questionnaire-Gynaecological Cancer Module, M months, *N* = number of women at risk at the specific timepoint, *n* = number of sexually active women at risk at the specific timepoint according to EORTC QLQ-CX24 item 19, O = observed number of women at the specific timepoint.

Figure 3I-III shows that the majority of the women in both groups had no physician-reported vaginal stenosis, dryness, and dyspareunia during follow-up, followed by grade 1 (not interfering with sexual functioning). Grade 1 vaginal stenosis was seen in 8 (7.5%, intervention group) and 4 (3%, care-as-usual group) women at baseline (before radiotherapy), gradually increasing to 21 (24%) women in the intervention group and 20 (23%) women in the care-as-usual group at 12 months after radiotherapy. Grade 1 vaginal dryness was reported in 2 (2%, intervention group) and 4 (4%, care-as-usual group) women before radiotherapy, increasing to 18 (21%) and 26 (32%) women, respectively, at 12 months after radiotherapy. Grade 1 dyspareunia was assessed in 3 (4%, intervention group) and 10 (12%, care-as-usual group) women before radiotherapy, increasing to 24 (29%) and 14 (20%) women, respectively, at 12 months after radiotherapy. There was no clear increase or decrease in patient-reported feelings of vaginal shortness, dryness and pain during intercourse in the follow-up period (Fig. 3IV–VI), with most sexually active women reporting ‘none’ or ‘a little’ (respectively, 61 (87%), 55 (78.5%) and 64 (91.5%) in the intervention group and 54 (87%), 54 (87%) and 58 (93.5%) in the care-as-usual group at 12 months after radiotherapy). At 12 months, more substantial (‘quite a bit’ or ‘very much’) feelings of vaginal shortness, dryness and pain during intercourse were reported by 9 (13%), 15 (21%) and 6 (9%) sexually active women in the intervention group versus 8 (13%), 8 (13%), and 4 (6.5%) sexually active women in the care-as-usual group, respectively. See Supplementary Appendixes 4 and 5 (p. 15–16) for additional physician and patient-reported outcomes on vaginal functioning.

**Fig. 4:**
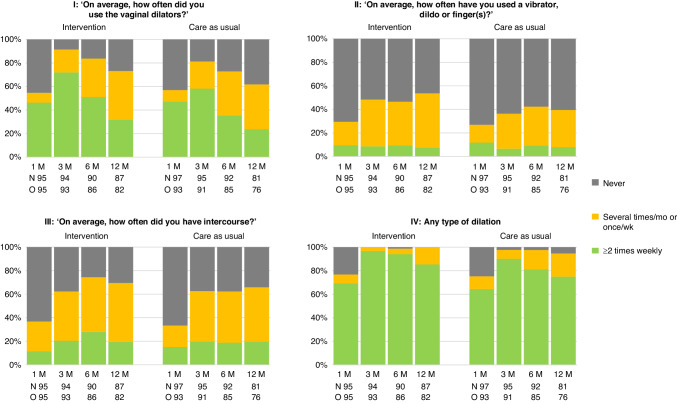
Patient-reported dilation in the past 4 weeks over time. The proportion of women is shown in percentages. M months, mo month, *N* = number of women at risk who received dilators, O = observed number of women at the specific timepoint, wk week.

Figure 4 shows the patient-reported dilation by the arm for women in the external beam radiotherapy combined with the brachytherapy group. Any type of dilation used at least two times per week, including dilators, vibrators, dildos, fingers or intercourse combined, that was employed by women who received brachytherapy, was reported by 66 (69.5%) women in the intervention group and 60 (64.5%) women in the care-as-usual group at 1 month after radiotherapy, increasing to 90 (97%) women and 82 (90%) women at 3 months after radiotherapy, respectively (Fig. 4IV). Any type of dilation ≥2 times per week slightly decreased to 70 (85%) women in the intervention group, and 57 (75%) women in the care-as-usual group at 12 months after radiotherapy.

Likelihood ratio tests indicated no significant improvement in model fit when including the treatment group (*P* > 0.05). No differences in FSFI total scores were found between the groups at any timepoint (*P* = 0.37). This suggests that the sexual rehabilitation intervention had no significant impact on better sexual functioning compared to the care-as-usual group. This result applied to most (95%) of the other outcomes (for more details, see Supplementary Appendix 3, p. 3–14).

## Discussion

The SPARC trial is to our knowledge the first robustly powered randomised trial to investigate the efficacy of a nurse-led sexual rehabilitation intervention to improve sexual recovery and compliance with dilator use for women treated with radiotherapy and brachytherapy for gynaecological cancers. Contrary to expectations based on the pilot study [14], this trial did not show a significant benefit of the nurse-led sexual rehabilitation intervention over care-as-usual in improving sexual functioning, dilator compliance, and reducing sexual distress and vaginal symptoms, 1 year after radiotherapy. However, compared to previous data on similar treatments, women in both study groups had relatively high rates of sexual activity, with overall 70% reporting to be sexually active at 12 months, compared to 40–50% in previous studies [1, 2, 4]. Most women in both study groups had no or little physician-reported vaginal stenosis, with most sexually active women reporting no or only a little feeling of vaginal shortness, dryness and pain during intercourse at 12 months after radiotherapy. Substantial vaginal and sexual functioning problems were rare. Also, any dilation ≥2 times weekly, including dilators, vibrators, dildos, fingers or intercourse combined, was reported by 85% of the participants in the intervention group and by 75% of the participants in the care-as-usual group at 12 months, similar to the pilot study [14]. Almost half of the women in both groups continued to experience clinical-level sexual distress even at the 12-month post-radiotherapy follow-up, indicating the complexity of sexual functioning.

The lack of notable differences in outcomes between the two groups is likely explained by the fact that both healthcare professionals in gynaecological oncology and patient advocacy groups have become more aware of the importance of early sexual rehabilitation care and associated issues since the start of the trial. Since the completion of the pilot study [14], standard sexual rehabilitation care in the Netherlands already involved a one-month post-radiotherapy appointment with a physician or nurse. During this appointment, women received comprehensive verbal and written information on sexual rehabilitation. In addition, a vaginal dilator set with lubrication gel was provided free of charge to women who had undergone pelvic radiotherapy with brachytherapy, along with explicit guidance on use, both within and outside of trial participation. The comprehensive training of the nurses conducting the intervention in the SPARC trial led to further in-depth knowledge, and to increased skills in informing and coaching and addressing specific personal issues and questions for these women across all centres, including in most centres those randomised to the care-as-usual group. In addition, during the study, the Dutch patient advocacy group for women with gynaecological cancers (Olijf) developed a specialised patient website focused on sexual rehabilitation [23]. The website’s widespread visibility and accessible information on sexuality and post-treatment rehabilitation were shared with all trial participants, fostering informal discussions among patients in online forums and with caregivers, both at the treatment centres and beyond.

To gain a more comprehensive understanding of the coaching and information of the women randomised to the care-as-usual group, we incorporated post hoc questionnaires for the centres about their standard care. The responses revealed that six out of ten centres had implemented additional improvements to standard sexual rehabilitation care during the study period, and that half of the centres had arranged at least one additional care pathway appointment within the first year after radiotherapy, with a specific focus on sexuality. Moreover, all centres ensured that sexuality remained a standard topic in all follow-up appointments with physicians. As the sexual rehabilitation sessions with the nurse were directly scheduled after these appointments, sexuality was discussed at the same timepoints in both study groups.

Women with cervical cancer constituted the large majority (88%) of the current study population, making our study outcomes particularly relevant for these relatively young women treated with intensive combined chemoradiotherapy and brachytherapy. The trial’s guidelines on sexual rehabilitation align with recent guidelines that advocate for heightened attention to post-radiotherapy sexual functioning in gynaecological cancer patients, including those treated with pelvic radiotherapy alone, albeit with more emphasis on rehabilitation after treatment in general and less on vaginal dilation [24, 25]. Considering that nurses can devote more time to patient interaction, are often easily accessible for patients, and can integrate their role in information and counselling in other clinical tasks, their role would be a cost-effective strategy for dedicated sexual rehabilitation. The cost-effectiveness of the nurse-led intervention versus care-as-usual is topic of subsequent analysis.

This trial has several notable strengths, including the well-powered randomised trial design, the participation of all Dutch gynaecological oncology centres, a limited dropout of study participants, the use of a clear treatment protocol and extensive training protocol, including an adherence and competency assessment by an independent panel, and the invitation to the women’s partners to join the intervention sessions. This study also has some limitations. First, as it turned out, the improvements in standard sexual rehabilitation after completion of radiation therapy may have unintentionally impacted on care of the care-as-usual group. Despite instructions to physicians and nurses to manage both study groups differently, their involvement with both study groups may have resulted similar initial post-radiotherapy psychosexual care. This well-known problem of contamination within individually randomised intervention studies could have been avoided by cluster randomisation (i.e., on the centre level instead of the patient level). However, this method also introduces other potential threats to internal validity, as the number of centres in our study is limited and only a part of the centres could be randomised (*n* = 8, as a consequence of the training that was already completed in two centres for the pilot study) [14]. Because of the specific variation in the patient population, radiotherapy treatment procedures and follow-up procedures across centres, we decided to randomise on the patient level. Furthermore, the FSFI, which is widely employed to evaluate sexual functioning in female cancer survivors, could yield biased results for sexually inactive women due to lack of a partner, relationship quality, or reasons unrelated to cancer treatment effects [17, 26]. To mitigate this, we randomised participants with stratification based on partner status, and included the response option ‘not applicable, no partner’ for items concerning the partner relationship, thereby minimising potential imbalance in the study outcomes. It is also possible that sexual functioning may further improve on the longer term. To investigate potential further recovery and to assess if the high rates of sexual activity will be sustained over time, a long-term evaluation at 24 months after radiotherapy was added per protocol amendment and results will be available next year. Finally, it could be argued that this study attracted relatively young and motivated participants, and that the improvement in vaginal and sexual functioning in both study groups was due to recovery over time. However, prospective studies involving cohorts of women treated with radio(chemo)therapy and brachytherapy for advanced or recurrent cervical cancer, without any standard sexual rehabilitation care, reported clearly higher prevalence rates of feelings of vaginal shortness, dryness and pain during sexual activity, vaginal stenosis and lower compliance with dilator use [4, 7, 8, 27, 28].

The prospective EMBRACE vaginal morbidity sub-study, which recommended sexual rehabilitation counselling after treatment, along with clear instructions on dilator use, demonstrated similar outcomes to our study regarding stenosis (physician-reported), vaginal shortness, dryness, and dyspareunia (patient-reported) [29]. Half of sexually active women reported feelings of vaginal shortness, dryness, and intercourse-related pain, which was also the case in our study cohort, although in vast majority rated as ‘a little’. However, in the EMBRACE vaginal morbidity sub-study cohort 54% reported to be sexually active at 12 months, while this was ~70% in the SPARC trial, possibly reflecting the effects of the increased patient education and awareness on early sexual rehabilitation. Regarding vaginal stenosis, either no or only mild stenosis was found at 12 months in both study cohorts, probably resulting from the improved radiotherapy and brachytherapy techniques causing less severe vaginal effects than in historical cohorts [30] along with more frequent vaginal dilation reported by the participants. In the SPARC trial, 85% (intervention) versus 75% (care-as-usual) of women who received brachytherapy, reported using any form of dilation at least twice a week at 12 months, indicating high compliance.

The results of the SPARC trial highlight the improved sexual rehabilitation care for women undergoing intensive radio(chemo)therapy and brachytherapy in the Netherlands, both before and during the study period, emphasising the importance of awareness, education and comprehensive care. This may have resulted in comparable sexual rehabilitation for both study groups. We therefore regard this care-as-usual approach as best practice to improve sexuality and thus quality-of-life after gynaecological cancer treatment. This approach encompasses thorough patient information, as well as a sexual rehabilitation appointment with a specifically trained dedicated nurse at 1 month post-radiotherapy, including explicit guidance on dilator use and coaching on resuming sexual activities for women who underwent radiotherapy combined with brachytherapy, and dedicated follow-up regarding sexual functioning and dilator use over the first year after completion of treatment.

### Supplementary information

Supplementary Materials Appendix 1-6 SPARC-trial

## Data Availability

All (Dutch) materials, such as information booklets and the treatment protocol, will be available by the end of the current study after the publication of the results. Data containing potentially identifying or sensitive patient information are restricted according to European law (General Data Protection Regulation (GDPR)). The datasets generated and/or analysed during the current study are not available in a public repository but are available upon reasonable request after evaluation of a dedicated study protocol via MtK (M.M.ter_Kuile@lumc.nl).
