# Supplementary material for: Efficacy of a nurse-led sexual rehabilitation intervention for women with gynaecological cancers receiving radiotherapy: results of a randomised trial
Source: Br J Cancer. 2024 Jul 3;131(5):808–19. doi: 10.1038/s41416-024-02775-8 (PMC11369252; doi:10.1038/s41416-024-02775-8)
Supplement: Supplementary file 1 — Supplementary Materials Appendix 1-6 SPARC-trial [file 41416_2024_2775_MOESM1_ESM.pdf]

# Supplementary Materials

**Supplementary appendix 1 Participating centres including study teams**

| Centre | Principle Investigator (and Associated Investigators) | Nurses | Supervisors |
|---|---|---|---|
| Amsterdam Medical Center | Henrike Westerveld | Wilma van der Ham-Paalman<br>Sandra Diergaarde<br>Wendy Lesterhuis | Ellen Laan†<br>Hanneke Termeer |
| Catharina Hospital | Jeltsje Cnossen<br>Hetty van den Berg | Noor Vincent<br>Ivonne Oomen | Rian Brus<br>Simone Speelman |
| Erasmus Medical Center | Jan Willem Mens<br>Helena van Doorn<br>Remi Nout | Claudia Mangelaars<br>Karin Dupree<br>Nicoline Schuur – van ’t Hof<br>Sabine Blom<br>Christel de Jager-Blom | Hanneke Bolt |
| Leiden University Medical Center | Carien Creutzberg<br>Cor de Kroon<br>Laura Velema | Corine de Jong<br>Mirjam Laman | Charlotte Tuijnman-Raasveld<br>Ellen de Groot |
| Maastro | Ludy Lutgens | Moniek Kamps<br>Charlotte Penders | Reinhilde Melles |
| Netherlands Cancer Institute | Marlies Nowee | Kirsten de Greef<br>Erin Gardebroek-de Boer | Ilaniek Zantingh |
| Radboud University Medical Center | An Snyers | Annemieke Janssens<br>Lotte Knapen | Marianne Vergeer |
| Radiotherapiegroep | Dorien Haverkort | Janine de Neeff<br>Truus Kroeze | Irma Bosman |
| University Medical Center Groningen | Jannet Beukema | Bianca Kramer-Medema<br>Nina van Linde | Ria Bosgraaf |
| University Medical Center Utrecht | Ina Jurgenliemk-Schulz | Nicole van den Berg<br>Catharina van der Linden-Hunziker<br>Ankie Krol Veraar | Ilaniek Zantingh |

**Supplementary appendix 2 Additional patient and physician-reported outcomes**

| Variable | Questionnaire | Clinical cut-off score |
|---|---|---|
| Sexual functioning | FSFI | ≤26·55 |
| Sexual desire | FSFI | NA |
| Sexual arousal | FSFI | NA |
| Vaginal lubrication | FSFI | NA |
| Orgasmic function | FSFI | NA |
| Sexual satisfaction | FSFI | NA |
| Sexual pain | FSFI | NA |
| Sexual distress | FSDS | ≥15 |
| Symptom experience | EORTC QLQ-CX24 | NA |
| Body image | EORTC QLQ-CX24/QLQ-EN24 | NA |
| Sexual/vaginal functioning (dryness, shortness, tightness, pain during intercourse) | EORTC QLQ-CX24/QLQ-EN24 | NA |
| Vaginal tightness | EORTC QLQ-CX24/QLQ-EN24 | NA |
| Lymphedema | EORTC QLQ-CX24/QLQ-EN24 | NA |
| Peripheral neuropathy | EORTC QLQ-CX24 | NA |
| Menopausal symptoms | EORTC QLQ-CX24 | NA |
| Sexual worry | EORTC QLQ-CX24 | NA |
| Sexual activity | EORTC QLQ-CX24/QLQ-EN24 | NA |
| Sexual enjoyment | EORTC QLQ-CX24/QLQ-EN24 | NA |
| Urological symptoms | EORTC QLQ-EN24 | NA |
| Gastrointestinal symptoms | EORTC QLQ-EN24/QLQ-EN24 | NA |
| Sexual interest | EORTC QLQ-EN24 | NA |
| Fear of non-penetration sexual activity | FSQ | NA |
| Fear of coitus/vaginal penetration | FSQ | NA |
| Relationship dissatisfaction | MMQ | ≥20 |
| Psychological distress | HADS | ≥14 |
| Depression | HADS | ≥7 |
| Anxiety | HADS | ≥7 |
| Physical function | EORTC QLQ-C30 | NA |
| Role function | EORTC QLQ-C30 | NA |
| Emotional function | EORTC QLQ-C30 | NA |
| Cognitive function | EORTC QLQ-C30 | NA |
| Social function | EORTC QLQ-C30 | NA |
| Fatigue | EORTC QLQ-C30 | NA |
| Nausea/vomiting | EORTC QLQ-C30 | NA |
| Pain | EORTC QLQ-C30 | NA |
| Dyspnoea | EORTC QLQ-C30 | NA |
| Sleep disturbance | EORTC QLQ-C30 | NA |
| Appetite loss | EORTC QLQ-C30 | NA |
| Constipation | EORTC QLQ-C30 | NA |
| Diarrhoea | EORTC QLQ-C30 | NA |
| Financial impact | EORTC QLQ-C30 | NA |
| Global QoL | EORTC QLQ-C30 | NA |
| Treatment related stress – intrusion | IES | NA |
| Treatment related stress – avoidance | IES | NA |
| Cost-effectiveness | | |
| Sexual health care use | - | NA |
| Quality of life | EQ-5D-5L | NA |
| Credibility of analogue therapy rationales | CEQ | NA |
| Physician-reported Bleeding | CTCAE | ≥grade 3 = severe |
| Physician-reported Mucositis | CTCAE | ≥grade 3 = severe |
| Physician-reported Discharge | CTCAE | ≥grade 3 = severe |
| Physician-reported Fibrosis | CTCAE | ≥grade 3 = severe |
| Physician-reported Atrophy/telangiectasia | CTCAE | ≥grade 3 = severe |
| Physician-reported Pain | CTCAE | ≥grade 3 = severe |
| Physician-reported Dyspareunia | CTCAE | ≥grade 3 = severe |
| Physician-reported vaginal estradiol/estriol | CRF | NA |
| Physician-reported hormone replacement therapy | CRF | NA |

*Note.* CEQ = Credibility/Expectancy Questionnaire; CRF = Case Report Form; CTCAE = Common Terminology Criteria for Adverse Events; EORTC QLQ-C30 = European Organization for Research and Treatment of Cancer Quality of Life Questionnaire-Core 30; EORTC QLQ-CX24 = European Organization for Research and Treatment of Cancer Quality of Life Questionnaire-Gynaecological Cancer Module; EORTC QLQ-EN24 = European Organization for Research and Treatment of Cancer Quality of Life Questionnaire-Endometrial Cancer Module; EQ-5D-5L = EuroQol 5D-5L; FSDS = Female Sexual Distress Scale; FSFI = Female Sexual Function Index; FSQ = Fear of Sexuality Questionnaire; HADS = Hospital Anxiety and Depression Scale; IES = Impact of Event Scale; MMQ = Maudsley Marital Questionnaire; NA = not applicable

**Supplementary appendix 3 Total and subscale mean values at retrospective baseline (for FSFI and FSDS) 1, 3, 6 and 12 months after radiotherapy and GLMM likelihood ratio test outcomes of primary and secondary continuous outcome measures**

| | Assessment | | | | | | | | | | | | | | | GLMM outcomes | |
|---|---|---|---|---|---|---|---|---|---|---|---|---|---|---|---|---|---|
| | T1 | | | T2 | | | T3 | | | T4 | | | T5 | | | | |
| Outcome measure (range score) | N | M/% | SD | N | M/% | SD | N | M/% | SD | N | M/% | SD | N | M/% | SD | LRT(df) | P (overall model) |
| **Primary** | | | | | | | | | | | | | | | | | |
| Sexual functioning | | | | | | | | | | | | | | | | | |
| FSFI total (2-36) | | | | | | | | | | | | | | | | 10·88(10) | 0·37 |
| Intervention | 112 | 28·08 | 6·48 | 108 | 18·36 | 10·27 | 106 | 21·98 | 9·45 | 98 | 23·82 | 8·83 | 94 | 22·57 | 9·30 | | |
| Care-as-usual | 117 | 28·30 | 7·07 | 110 | 17·38 | 10·64 | 106 | 22·26 | 9·10 | 98 | 22·89 | 8·74 | 87 | 21·76 | 10·42 | | |
| Sexual desire | | | | | | | | | | | | | | | | | |
| FSFI sexual desire (1·2-6) | | | | | | | | | | | | | | | | 7·59(10) | 0·67 |
| Intervention | 112 | 3·81 | 0·99 | 108 | 2·92 | 1·25 | 106 | 3·05 | 1·27 | 99 | 3·31 | 1·30 | 94 | 2·99 | 1·32 | | |
| Care-as-usual | 117 | 3·76 | 0·99 | 110 | 2·80 | 1·24 | 106 | 2·99 | 1·23 | 98 | 3·04 | 1·18 | 88 | 3·08 | 1·34 | | |
| Sexual arousal | | | | | | | | | | | | | | | | | |
| FSFI arousal (0-6) | | | | | | | | | | | | | | | | 13·16(10) | 0·21 |
| Intervention | 112 | 4·75 | 1·40 | 108 | 2·93 | 2·09 | 106 | 3·63 | 1·95 | 98 | 3·90 | 1·89 | 94 | 3·52 | 1·98 | | |
| Care-as-usual | 117 | 4·81 | 1·35 | 110 | 2·72 | 2·31 | 106 | 3·67 | 1·90 | 98 | 3·75 | 1·91 | 88 | 3·69 | 2·07 | | |
| Vaginal lubrication | | | | | | | | | | | | | | | | | |
| FSFI lubrication (0-6) | | | | | | | | | | | | | | | | 4·06(10) | 0·95 |
| Intervention | 112 | 5·14 | 1·49 | 108 | 3·10 | 2·43 | 106 | 3·76 | 2·19 | 98 | 4·15 | 2·11 | 94 | 3·88 | 2·23 | | |
| Care-as-usual | 117 | 5·24 | 1·52 | 110 | 2·89 | 2·52 | 106 | 3·93 | 2·14 | 98 | 4·17 | 2·13 | 88 | 3·80 | 2·40 | | |
| Orgasmic function | | | | | | | | | | | | | | | | | |
| FSFI orgasm (0-6) | | | | | | | | | | | | | | | | 6·52(10) | 0·77 |
| Intervention | 112 | 4·80 | 1·32 | 108 | 3·15 | 2·35 | 106 | 3·88 | 2·10 | 98 | 4·05 | 1·91 | 94 | 3·95 | 1·98 | | |
| Care-as-usual | 117 | 4·97 | 1·54 | 110 | 2·98 | 2·49 | 106 | 4·03 | 2·11 | 98 | 4·19 | 2·11 | 88 | 3·84 | 2·32 | | |
| Sexual satisfaction | | | | | | | | | | | | | | | | | |
| FSFI satisfaction (0-6) | | | | | | | | | | | | | | | | 1·49(10) | 0·99 |
| Intervention | 112 | 4·88 | 1·19 | 108 | 3·77 | 1·54 | 106 | 4·11 | 1·59 | 98 | 4·40 | 1·35 | 94 | 4·18 | 1·53 | | |
| Care-as-usual | 117 | 4·77 | 1·36 | 110 | 3·62 | 1·58 | 106 | 4·11 | 1·52 | 98 | 4·20 | 1·49 | 87 | 4·06 | 1·54 | | |
| Sexual pain | | | | | | | | | | | | | | | | | |
| FSFI pain (0-6) | | | | | | | | | | | | | | | | 10·75(10) | 0·37 |
| Intervention | 112 | 4·70 | 1·82 | 108 | 2·49 | 2·52 | 106 | 3·54 | 2·28 | 98 | 4·02 | 2·08 | 94 | 4·03 | 2·25 | | |
| Care-as-usual | 117 | 4·75 | 1·89 | 110 | 2·37 | 2·45 | 106 | 3·54 | 2·28 | 98 | 3·54 | 2·18 | 87 | 3·38 | 2·52 | | |

**Supplementary appendix 3** *Continued*

| | Assessment | | | | | | | | | | | | | | | GLMM outcomes | |
|---|---|---|---|---|---|---|---|---|---|---|---|---|---|---|---|---|---|
| | T1 | | | T2 | | | T3 | | | T4 | | | T5 | | | | |
| Outcome measure (range score) | N | M/ % | SD | N | M/ % | SD | N | M/ % | SD | N | M/ % | SD | N | M/ % | SD | LRT(df) | P (overall model) |
| Sexual activity | | | | | | | | | | | | | | | | | |
| Intervention | | | | | | | | | | | | | | | | 0·54(5) | 0·99 |
| *Never* | 20 | 18·0 | ·· | 45 | 41·7 | ·· | 36 | 34·0 | ·· | 34 | 34·7 | ·· | 27 | 28·7 | ·· | | |
| *Several times per month* | 33 | 29·7 | ·· | 26 | 24·1 | ·· | 32 | 30·2 | ·· | 29 | 29·6 | ·· | 36 | 38·3 | ·· | | |
| *1 time/week* | 20 | 18·0 | ·· | 17 | 15·7 | ·· | 17 | 16·0 | ·· | 16 | 16·3 | ·· | 15 | 16·0 | ·· | | |
| *≥2 times/week* | 38 | 34·2 | ·· | 20 | 18·5 | ·· | 21 | 19·8 | ·· | 19 | 19·4 | ·· | 16 | 17·0 | ·· | | |
| Care-as-usual | | | | | | | | | | | | | | | | | |
| *Never* | 22 | 19·0 | ·· | 45 | 40·9 | ·· | 37 | 34·9 | ·· | 34 | 34·7 | ·· | 27 | 31·0 | ·· | | |
| *Several times per month* | 37 | 31·9 | ·· | 28 | 25·5 | ·· | 34 | 32·1 | ·· | 37 | 37·8 | ·· | 37 | 42·5 | ·· | | |
| *1 time/week* | 28 | 24·1 | ·· | 11 | 10·0 | ·· | 16 | 15·1 | ·· | 11 | 11·2 | ·· | 11 | 12·6 | ·· | | |
| *≥2 times/week* | 29 | 25·0 | ·· | 26 | 23·6 | ·· | 19 | 17·9 | ·· | 16 | 16·3 | ·· | 12 | 13·8 | ·· | | |
| Sexual Intercourse | | | | | | | | | | | | | | | | | |
| Intervention | | | | | | | | | | | | | | | | 3·36(3) | 0·34 |
| *Never* | 15 | 13·6 | ·· | 62 | 57·9 | ·· | 37 | 34·9 | ·· | 25 | 25·5 | ·· | 29 | 30·9 | ·· | | |
| *Several times per month* | 33 | 30·0 | ·· | 19 | 17·8 | ·· | 24 | 22·6 | ·· | 28 | 28·6 | ·· | 22 | 23·4 | ·· | | |
| *1 time/week* | 18 | 16·4 | ·· | 14 | 13·1 | ·· | 22 | 20·8 | ·· | 20 | 20·4 | ·· | 26 | 27·7 | ·· | | |
| *≥2 times/week* | 44 | 40·0 | ·· | 12 | 11·2 | ·· | 23 | 21·7 | ·· | 25 | 25·5 | ·· | 17 | 18·1 | ·· | | |
| Care-as-usual | | | | | | | | | | | | | | | | | |
| *Never* | 20 | 17·1 | ·· | 71 | 64·5 | ·· | 37 | 34·9 | ·· | 37 | 37·8 | ·· | 31 | 35·6 | ·· | | |
| *Several times per month* | 37 | 31·6 | ·· | 13 | 11·8 | ·· | 32 | 30·2 | ·· | 26 | 26·5 | ·· | 25 | 28·7 | ·· | | |
| *1 time/week* | 25 | 21·4 | ·· | 7 | 6·4 | ·· | 17 | 16·0 | ·· | 13 | 13·3 | ·· | 13 | 14·9 | ·· | | |
| *≥2 times/week* | 35 | 29·9 | ·· | 19 | 17·3 | ·· | 20 | 18·9 | ·· | 22 | 22·4 | ·· | 18 | 20·7 | ·· | | |
| **Secondary** | | | | | | | | | | | | | | | | | |
| Sexual Distress | | | | | | | | | | | | | | | | | |
| FSDS (0-48) | | | | | | | | | | | | | | | | 7·57(10) | 0·67 |
| Intervention | 112 | 9·32 | 9·40 | 107 | 14·37 | 10·49 | 106 | 13·69 | 10·55 | 98 | 14·17 | 10·31 | 94 | 14·65 | 12·07 | | |
| Care-as-usual | 117 | 8·74 | 9·36 | 110 | 12·95 | 10·01 | 106 | 13·17 | 9·92 | 98 | 14·35 | 11·36 | 87 | 13·94 | 10·97 | | |

**Supplementary appendix 3** *Continued*

| | Assessment | | | | | | | | | | | | | | | GLMM outcomes | |
|---|---|---|---|---|---|---|---|---|---|---|---|---|---|---|---|---|---|
| | T1 | | | T2 | | | T3 | | | T4 | | | T5 | | | | |
| Outcome measure (range score) | N | M/ % | SD | N | M/ % | SD | N | M/ % | SD | N | M/ % | SD | N | M/ % | SD | LRT(df) | P (overall model) |
| Vaginal dilation | | | | | | | | | | | | | | | | | |
| Dilator use | | | | | | | | | | | | | | | | | |
| Intervention | | | | | | | | | | | | | | | | 5·25(4) | 0·24 |
| *Never* | ·· | ·· | ·· | 43 | 45·3 | ·· | 8 | 8·6 | ·· | 14 | 16·3 | ·· | 22 | 26·8 | ·· | | |
| *Several times per month* | ·· | ·· | ·· | 3 | 3·2 | ·· | 6 | 6·5 | ·· | 8 | 9·3 | ·· | 19 | 23·2 | ·· | | |
| *1 time/week* | ·· | ·· | ·· | 5 | 5·3 | ·· | 12 | 12·9 | ·· | 20 | 23·3 | ·· | 15 | 18·3 | ·· | | |
| *2 times/week* | ·· | ·· | ·· | 10 | 10·5 | ·· | 27 | 29·0 | ·· | 16 | 18·6 | ·· | 17 | 20·7 | ·· | | |
| *3 times/week* | ·· | ·· | ·· | 29 | 30·5 | ·· | 38 | 40·9 | ·· | 25 | 29·1 | ·· | 9 | 11·0 | ·· | | |
| *4-6 times/week* | ·· | ·· | ·· | 5 | 5·3 | ·· | 2 | 2·2 | ·· | 3 | 3·5 | ·· | 0 | 0 | ·· | | |
| *Daily* | ·· | ·· | ·· | 0 | 0 | ·· | 0 | 0 | ·· | 0 | 0 | ·· | 0 | 0 | ·· | | |
| Care-as-usual | | | | | | | | | | | | | | | | | |
| *Never* | ·· | ·· | ·· | 40 | 43·0 | ·· | 17 | 18·7 | ·· | 23 | 27·1 | ·· | 29 | 38·2 | ·· | | |
| *Several times per month* | ·· | ·· | ·· | 4 | 4·3 | ·· | 12 | 13·2 | ·· | 13 | 15·3 | ·· | 23 | 30·3 | ·· | | |
| *1 time/week* | ·· | ·· | ·· | 5 | 5·4 | ·· | 9 | 9·9 | ·· | 19 | 22·4 | ·· | 6 | 7·9 | ·· | | |
| *2 times/week* | ·· | ·· | ·· | 16 | 17·2 | ·· | 21 | 23·1 | ·· | 15 | 17·6 | ·· | 10 | 13·2 | ·· | | |
| *3 times/week* | ·· | ·· | ·· | 25 | 26·9 | ·· | 29 | 31·9 | ·· | 13 | 15·3 | ·· | 6 | 7·9 | ·· | | |
| *4-6 times/week* | ·· | ·· | ·· | 1 | 1·1 | ·· | 3 | 3·3 | ·· | 1 | 1·2 | ·· | 2 | 2·6 | ·· | | |
| *Daily* | ·· | ·· | ·· | 2 | 2·2 | ·· | 0 | 0 | ·· | 1 | 1·2 | ·· | 0 | 0 | ·· | | |
| Vaginal dilation | | | | | | | | | | | | | | | | | |
| Vibrator/dildo /fingers | | | | | | | | | | | | | | | | 1·90(4) | 0·75 |
| Intervention | ·· | ·· | ·· | 67 | 70·5 | ·· | 48 | 51·6 | ·· | 46 | 53·5 | ·· | 38 | 46·3 | ·· | | |
| *Never* | ·· | ·· | ·· | 12 | 12·6 | ·· | 25 | 26·9 | ·· | 21 | 24·4 | ·· | 28 | 34·1 | ·· | | |
| *Several times* | | | | | | | | | | | | | | | | | |
| *per month* | ·· | ·· | ·· | 7 | 7·4 | ·· | 12 | 12·9 | ·· | 11 | 12·8 | ·· | 10 | 12·2 | ·· | | |
| *1 time/week* | ·· | ·· | ·· | 4 | 4·2 | ·· | 6 | 6·5 | ·· | 6 | 7·0 | ·· | 2 | 2·4 | ·· | | |
| *2 times/week* | ·· | ·· | ·· | 4 | 4·2 | ·· | 2 | 2·2 | ·· | 2 | 2·3 | ·· | 4 | 4·9 | ·· | | |
| *3 times/week* | ·· | ·· | ·· | 1 | 1·1 | ·· | 0 | 0 | ·· | 0 | 0 | ·· | 0 | 0 | ·· | | |
| *4-6 times/week* | ·· | ·· | ·· | 0 | 0 | ·· | 0 | 0 | ·· | 0 | 0 | ·· | 0 | 0 | ·· | | |
| *Daily* | | | | | | | | | | | | | | | | | |
| Care-as-usual | ·· | ·· | ·· | 68 | 73·1 | ·· | 58 | 63·7 | ·· | 49 | 57·6 | ·· | 46 | 60·5 | ·· | | |
| *Never* | ·· | ·· | ·· | 12 | 12·9 | ·· | 21 | 23·1 | ·· | 23 | 27·1 | ·· | 19 | 25·0 | ·· | | |
| *Several times* | | | | | | | | | | | | | | | | | |
| *per month* | ·· | ·· | ·· | 2 | 2·2 | ·· | 6 | 6·6 | ·· | 5 | 5·9 | ·· | 5 | 6·6 | ·· | | |
| *1 time/week* | ·· | ·· | ·· | 7 | 7·5 | ·· | 2 | 2·2 | ·· | 2 | 2·4 | ·· | 5 | 6·6 | ·· | | |
| *2 times/week* | ·· | ·· | ·· | 2 | 2·2 | ·· | 2 | 2·2 | ·· | 3 | 3·5 | ·· | 1 | 1·3 | ·· | | |
| *3 times/week* | ·· | ·· | ·· | 1 | 1·1 | ·· | 1 | 1·1 | ·· | 2 | 2·4 | ·· | 0 | 0 | ·· | | |
| *4-6 times/week* | ·· | ·· | ·· | 1 | 1·1 | ·· | 1 | 1·1 | ·· | 1 | 1·2 | ·· | 0 | 0 | ·· | | |
| *Daily* | | | | | | | | | | | | | | | | | |

**Supplementary appendix 3** *Continued*

| | Assessment | | | | | | | | | | | | | | | GLMM outcomes | |
|---|---|---|---|---|---|---|---|---|---|---|---|---|---|---|---|---|---|
| | T1 | | | T2 | | | T3 | | | T4 | | | T5 | | | | |
| Outcome measure (range score) | N | M/ % | SD | N | M/ % | SD | N | M/ % | SD | N | M/ % | SD | N | M/ % | SD | LRT(df) | P (overall model) |
| Vaginal dilation - Intercourse | | | | | | | | | | | | | | | | | |
| Intervention | | | | | | | | | | | | | | | | 2·33(4) | 0·68 |
| *Never* | ·· | ·· | ·· | 60 | 63·2 | ·· | 35 | 37·6 | ·· | 22 | 25·6 | ·· | 25 | 30·5 | ·· | | |
| *Several times per month* | ·· | ·· | ·· | 10 | 10·5 | ·· | 18 | 19·4 | ·· | 24 | 27·9 | ·· | 18 | 22·0 | ·· | | |
| *1 time/week* | ·· | ·· | ·· | 14 | 14·7 | ·· | 21 | 22·6 | ·· | 16 | 18·6 | ·· | 23 | 28·0 | ·· | | |
| *2 times/week* | ·· | ·· | ·· | 9 | 9·5 | ·· | 13 | 14·0 | ·· | 16 | 18·6 | ·· | 11 | 13·4 | ·· | | |
| *3 times/week* | ·· | ·· | ·· | 2 | 2·1 | ·· | 5 | 5·4 | ·· | 6 | 7·0 | ·· | 3 | 3·7 | ·· | | |
| *4-6 times/week* | ·· | ·· | ·· | 0 | 0 | ·· | 0 | 0 | ·· | 1 | 1·2 | ·· | 2 | 2·4 | ·· | | |
| *Daily* | ·· | ·· | ·· | 0 | 0 | ·· | 1 | 1·1 | ·· | 1 | 1·2 | ·· | 0 | 0 | ·· | | |
| Care-as-usual | | | | | | | | | | | | | | | | | |
| *Never* | ·· | ·· | ·· | 62 | 66·7 | ·· | 34 | 37·4 | ·· | 32 | 37·6 | ·· | 26 | 34·2 | ·· | | |
| *Several times per month* | ·· | ·· | ·· | 12 | 12·9 | ·· | 27 | 29·7 | ·· | 28 | 32·9 | ·· | 23 | 30·3 | ·· | | |
| *1 time/week* | ·· | ·· | ·· | 5 | 5·4 | ·· | 12 | 13·2 | ·· | 9 | 10·6 | ·· | 12 | 15·8 | ·· | | |
| *2 times/week* | ·· | ·· | ·· | 6 | 6·5 | ·· | 9 | 9·9 | ·· | 10 | 11·8 | ·· | 8 | 10·5 | ·· | | |
| *3 times/week* | ·· | ·· | ·· | 5 | 5·4 | ·· | 6 | 6·6 | ·· | 4 | 4·7 | ·· | 3 | 3·9 | ·· | | |
| *4-6 times/week* | ·· | ·· | ·· | 2 | 2·2 | ·· | 2 | 2·2 | ·· | 1 | 1·2 | ·· | 3 | 3·9 | ·· | | |
| *Daily* | ·· | ·· | ·· | 1 | 1·1 | ·· | 1 | 1·1 | ·· | 1 | 1·2 | ·· | 1 | 1·3 | ·· | | |
| Any type of vaginal dilation ≥2 times/week | | | | | | | | | | | | | | | | | |
| Intervention | | | | | | | | | | | | | | | | 1·39(4) | 0·85 |
| *Yes* | ·· | ·· | ·· | 66 | 69·5 | ·· | 90 | 96·8 | ·· | 81 | 94·2 | ·· | 70 | 85·4 | ·· | | |
| *No* | ·· | ·· | ·· | 29 | 30·5 | ·· | 3 | 3·2 | ·· | 5 | 5·8 | ·· | 12 | 14·6 | ·· | | |
| Care-as-usual | | | | | | | | | | | | | | | | | |
| *Yes* | ·· | ·· | ·· | 60 | 64·5 | ·· | 82 | 90·1 | ·· | 69 | 81·2 | ·· | 57 | 75·0 | ·· | | |
| *No* | ·· | ·· | ·· | 33 | 35·5 | ·· | 9 | 9·9 | ·· | 16 | 18·8 | ·· | 19 | 25·0 | ·· | | |
| Sexual functioning | | | | | | | | | | | | | | | | | |
| EORTC QLQ-CX24 (0-100) | | | | | | | | | | | | | | | | 10·64(8) | 0·22 |
| Intervention | ·· | ·· | ·· | 52 | 26·44 | 19·65 | 76 | 22·59 | 20·18 | 77 | 21·43 | 18·75 | 70 | 18·69 | 19·32 | | |
| Care-as-usual | ·· | ·· | ·· | 50 | 23·67 | 18·62 | 70 | 22·74 | 17·60 | 66 | 22·60 | 16·06 | 62 | 19·49 | 21·65 | | |
| Vaginal symptoms | | | | | | | | | | | | | | | | | |
| EORTC QLQ-CX24 (0-100) | | | | | | | | | | | | | | | | 3·92(8) | 0·86 |
| Intervention | ·· | ·· | ·· | 107 | 16·30 | 14·90 | 106 | 11·43 | 11·83 | 98 | 11·0 | 12·71 | 94 | 10·99 | 13·19 | | |
| Care-as-usual | ·· | ·· | ·· | 109 | 16·00 | 14·46 | 106 | 12·06 | 13·38 | 97 | 10·20 | 12·39 | 87 | 10·47 | 13·38 | | |

**Supplementary appendix 3** *Continued*

| Outcome measure (range score) | T1 N | T1 M/% | T1 SD | T2 N | T2 M/% | T2 SD | T3 N | T3 M/% | T3 SD | T4 N | T4 M/% | T4 SD | T5 N | T5 M/% | T5 SD | GLMM outcomes LRT(df) | GLMM outcomes P (overall model) |
|---|---|---|---|---|---|---|---|---|---|---|---|---|---|---|---|---|---|
| Sexual/vaginal functioning | | | | | | | | | | | | | | | | | |
| EORTC QLQ-CX24 | | | | | | | | | | | | | | | | | |
| Vaginal dryness | | | | | | | | | | | | | | | | | |
| Intervention | | | | | | | | | | | | | | | | 0·83(8) | 0·99 |
| *Not at all* | ·· | ·· | ·· | 20 | 38·5 | ·· | 35 | 46·1 | ·· | 35 | 45·5 | ·· | 36 | 51·4 | ·· | | |
| *A little* | ·· | ·· | ·· | 25 | 48·1 | ·· | 22 | 28·9 | ·· | 27 | 35·1 | ·· | 19 | 27·1 | ·· | | |
| *Quite a bit* | ·· | ·· | ·· | 5 | 9·6 | ·· | 11 | 14·5 | ·· | 12 | 15·6 | ·· | 11 | 15·7 | ·· | | |
| *Very much* | ·· | ·· | ·· | 2 | 3·8 | ·· | 8 | 10·5 | ·· | 3 | 3·9 | ·· | 4 | 5·7 | ·· | | |
| Care-as-usual | | | | | | | | | | | | | | | | | |
| *Not at all* | ·· | ·· | ·· | 25 | 50·0 | ·· | 30 | 42·9 | ·· | 26 | 39·4 | ·· | 31 | 50·0 | ·· | | |
| *A little* | ·· | ·· | ·· | 14 | 28·0 | ·· | 33 | 47·1 | ·· | 31 | 47·0 | ·· | 23 | 37·1 | ·· | | |
| *Quite a bit* | ·· | ·· | ·· | 9 | 18·0 | ·· | 3 | 4·3 | ·· | 7 | 10·6 | ·· | 4 | 6·5 | ·· | | |
| *Very much* | ·· | ·· | ·· | 2 | 4·0 | ·· | 4 | 5·7 | ·· | 2 | 3·0 | ·· | 4 | 6·5 | ·· | | |
| Sexual/vaginal functioning | | | | | | | | | | | | | | | | | |
| EORTC QLQ-CX24 | | | | | | | | | | | | | | | | | |
| vaginal shortness | | | | | | | | | | | | | | | | | |
| Intervention | | | | | | | | | | | | | | | | 1·18(8) | 0·99 |
| *Not at all* | ·· | ·· | ·· | 24 | 46·2 | ·· | 41 | 53·9 | ·· | 42 | 54·5 | ·· | 40 | 57·1 | ·· | | |
| *A little* | ·· | ·· | ·· | 21 | 40·4 | ·· | 22 | 28·9 | ·· | 20 | 26·0 | ·· | 21 | 30·0 | ·· | | |
| *Quite a bit* | ·· | ·· | ·· | 3 | 5·8 | ·· | 10 | 13·2 | ·· | 11 | 14·3 | ·· | 7 | 10·0 | ·· | | |
| *Very much* | ·· | ·· | ·· | 4 | 7·7 | ·· | 3 | 3·9 | ·· | 4 | 5·2 | ·· | 2 | 2·9 | ·· | | |
| Care-as-usual | | | | | | | | | | | | | | | | | |
| *Not at all* | ·· | ·· | ·· | 23 | 46·0 | ·· | 35 | 50·0 | ·· | 29 | 43·9 | ·· | 34 | 54·8 | ·· | | |
| *A little* | ·· | ·· | ·· | 21 | 42·0 | ·· | 27 | 38·6 | ·· | 28 | 42·4 | ·· | 20 | 32·3 | ·· | | |
| *Quite a bit* | ·· | ·· | ·· | 4 | 8·0 | ·· | 8 | 11·4 | ·· | 8 | 12·1 | ·· | 4 | 6·5 | ·· | | |
| *Very much* | ·· | ·· | ·· | 2 | 4·0 | ·· | 0 | 0 | ·· | 1 | 1·5 | ·· | 4 | 6·5 | ·· | | |
| Sexual/vaginal functioning | | | | | | | | | | | | | | | | | |
| EORTC QLQ-CX24 | | | | | | | | | | | | | | | | | |
| vaginal tightness | | | | | | | | | | | | | | | | | |
| Intervention | | | | | | | | | | | | | | | | 3·23(8) | 0·92 |
| *Not at all* | ·· | ·· | ·· | 22 | 42·3 | ·· | 47 | 61·8 | ·· | 54 | 70·1 | ·· | 51 | 72·9 | ·· | | |
| *A little* | ·· | ·· | ·· | 19 | 36·5 | ·· | 24 | 31·6 | ·· | 14 | 18·2 | ·· | 14 | 20·0 | ·· | | |
| *Quite a bit* | ·· | ·· | ·· | 7 | 13·5 | ·· | 5 | 6·6 | ·· | 8 | 10·4 | ·· | 5 | 7·1 | ·· | | |
| *Very much* | ·· | ·· | ·· | 4 | 7·7 | ·· | 0 | 0 | ·· | 1 | 1·3 | ·· | 0 | 0 | ·· | | |
| Care-as-usual | | | | | | | | | | | | | | | | | |
| *Not at all* | ·· | ·· | ·· | 25 | 50·0 | ·· | 33 | 47·1 | ·· | 38 | 57·6 | ·· | 39 | 62·9 | ·· | | |
| *A little* | ·· | ·· | ·· | 22 | 44·0 | ·· | 28 | 40·0 | ·· | 20 | 30·3 | ·· | 16 | 25·8 | ·· | | |
| *Quite a bit* | ·· | ·· | ·· | 1 | 2·0 | ·· | 7 | 10·0 | ·· | 8 | 12·1 | ·· | 4 | 6·5 | ·· | | |
| *Very much* | ·· | ·· | ·· | 2 | 4·0 | ·· | 2 | 2·9 | ·· | 0 | 0 | ·· | 3 | 4·8 | ·· | | |

**Supplementary appendix 3** *Continued*

| Outcome measure (range score) | Assessment | | | | | | | | | | | | | | | GLMM outcomes | |
|---|---|---|---|---|---|---|---|---|---|---|---|---|---|---|---|---|---|
| | T1 | | | T2 | | | T3 | | | T4 | | | T5 | | | | |
| | N | M/% | SD | N | M/% | SD | N | M/% | SD | N | M/% | SD | N | M/% | SD | LRT(df) | P (overall model) |
| Sexual/vaginal functioning | | | | | | | | | | | | | | | | | |
| EORTC QLQ-CX24 | | | | | | | | | | | | | | | | | |
| Pain during intercourse | | | | | | | | | | | | | | | | | |
| Intervention | | | | | | | | | | | | | | | | 1·24(8) | 0·99 |
| *Not at all* | ·· | ·· | ·· | 18 | 34·6 | ·· | 31 | 40·8 | ·· | 34 | 44·2 | ·· | 37 | 52·9 | ·· | | |
| *A little* | ·· | ·· | ·· | 29 | 55·8 | ·· | 38 | 50·0 | ·· | 35 | 45·5 | ·· | 27 | 38·6 | ·· | | |
| *Quite a bit* | ·· | ·· | ·· | 4 | 7·7 | ·· | 6 | 7·9 | ·· | 8 | 10·4 | ·· | 6 | 8·6 | ·· | | |
| *Very much* | ·· | ·· | ·· | 1 | 1·9 | ·· | 1 | 1·3 | ·· | 0 | 0 | ·· | 0 | 0 | ·· | | |
| Care-as-usual | | | | | | | | | | | | | | | | | |
| *Not at all* | ·· | ·· | ·· | 18 | 36·0 | ·· | 27 | 38·6 | ·· | 26 | 39·4 | ·· | 37 | 59·7 | ·· | | |
| *A little* | ·· | ·· | ·· | 28 | 56·0 | ·· | 38 | 54·3 | ·· | 35 | 53·0 | ·· | 21 | 33·9 | ·· | | |
| *Quite a bit* | ·· | ·· | ·· | 1 | 2·0 | ·· | 4 | 5·7 | ·· | 5 | 7·6 | ·· | 4 | 6·5 | ·· | | |
| *Very much* | ·· | ·· | ·· | 3 | 6·0 | ·· | 1 | 1·4 | ·· | 0 | 0 | ·· | 0 | 0 | ·· | | |
| Sexual worry | | | | | | | | | | | | | | | | | |
| EORTC QLQ-CX24 (0-100) | | | | | | | | | | | | | | | | 2·99(8) | 0·94 |
| Intervention | ·· | ·· | ·· | 107 | 39·25 | 32·96 | 105 | 26·98 | 28·53 | 98 | 21·43 | 27·60 | 94 | 22·34 | 29·89 | | |
| Care-as-usual | ·· | ·· | ·· | 109 | 37·00 | 32·18 | 106 | 25·47 | 25·43 | 98 | 24·15 | 30·57 | 87 | 19·92 | 28·96 | | |
| Sexual activity | | | | | | | | | | | | | | | | | |
| EORTC QLQ-CX24 (0-100) | | | | | | | | | | | | | | | | 8·89(8) | 0·35 |
| Intervention | ·· | ·· | ·· | 107 | 21·18 | 24·40 | 105 | 33·97 | 27·34 | 98 | 39·80 | 26·93 | 94 | 36·17 | 26·62 | | |
| Care-as-usual | ·· | ·· | ·· | 109 | 22·94 | 29·29 | 106 | 30·82 | 27·87 | 98 | 30·95 | 27·18 | 87 | 34·87 | 28·26 | | |
| Sexual enjoyment | | | | | | | | | | | | | | | | | |
| EORTC QLQ-CX24 (0-100) | | | | | | | | | | | | | | | | 11·58(8) | 0·17 |
| Intervention | ·· | ·· | ·· | 52 | 58·97 | 26·09 | 76 | 59·65 | 29·47 | 77 | 62·77 | 24·76 | 70 | 62·38 | 28·33 | | |
| Care-as-usual | ·· | ·· | ·· | 50 | 60·67 | 29·11 | 70 | 62·38 | 27·76 | 66 | 60·10 | 23·55 | 62 | 63·98 | 27·86 | | |
| Sexual interest | | | | | | | | | | | | | | | | | |
| EORTC QLQ-EN24 (0-100) | | | | | | | | | | | | | | | | 4·2(8) | 0·84 |
| Intervention | ·· | ·· | ·· | 107 | 30·84 | 27·36 | 105 | 34·92 | 27·50 | 98 | 38·78 | 27·78 | 94 | 34·04 | 29·32 | | |
| Care-as-usual | ·· | ·· | ·· | 109 | 30·28 | 25·88 | 106 | 34·91 | 27·36 | 98 | 35·71 | 24·99 | 87 | 34·48 | 28·05 | | |
| Symptom experience | | | | | | | | | | | | | | | | | |
| EORTC QLQ-CX24 (0-100) | | | | | | | | | | | | | | | | 3·01(8) | 0·93 |
| Intervention | ·· | ·· | ·· | 107 | 15·89 | 10·55 | 105 | 13·45 | 8·82 | 98 | 14·13 | 9·63 | 94 | 13·38 | 10·10 | | |
| Care-as-usual | ·· | ·· | ·· | 109 | 16·51 | 10·75 | 106 | 14·47 | 11·12 | 97 | 13·93 | 10·04 | 87 | 13·27 | 9·97 | | |
| Body image | | | | | | | | | | | | | | | | | |
| EORTC QLQ-CX24 (0-100) | | | | | | | | | | | | | | | | 4·17(8) | 0·84 |
| Intervention | ·· | ·· | ·· | 107 | 31·67 | 27·59 | 105 | 27·41 | 27·99 | 98 | 27·55 | 27·60 | 94 | 29·55 | 28·25 | | |
| Care-as-usual | ·· | ·· | ·· | 109 | 29·46 | 26·47 | 104 | 28·95 | 26·02 | 98 | 27·89 | 25·04 | 87 | 26·44 | 24·80 | | |

**Supplementary appendix 3** *Continued*

| | Assessment | | | | | | | | | | | | | | | GLMM outcomes | |
|---|---|---|---|---|---|---|---|---|---|---|---|---|---|---|---|---|---|
| | T1 | | | T2 | | | T3 | | | T4 | | | T5 | | | | |
| Outcome measure (range score) | N | M/ % | SD | N | M/ % | SD | N | M/ % | SD | N | M/ % | SD | N | M/ % | SD | LRT(df) | P (overall model) |
| Lymphedema | | | | | | | | | | | | | | | | | |
| EORTC QLQ-CX24 (0-100) | | | | | | | | | | | | | | | | 6·16(8) | 0·63 |
| Intervention | ·· | ·· | ·· | 107 | 11·22 | 21·45 | 106 | 11·32 | 21·52 | 98 | 14·97 | 26·70 | 94 | 13·83 | 23·14 | | |
| Care-as-usual | ·· | ·· | ·· | 109 | 8·26 | 19·85 | 106 | 11·01 | 23·33 | 98 | 10·88 | 21·29 | 87 | 15·33 | 27·28 | | |
| Peripheral neuropathy | | | | | | | | | | | | | | | | | |
| EORTC QLQ-CX24 (0-100) | | | | | | | | | | | | | | | | 2·68(6) | 0·85 |
| Intervention | ·· | ·· | ·· | 107 | 11·84 | 20·09 | 106 | 16·67 | 26·53 | 98 | 15·99 | 24·51 | 94 | 17·02 | 25·28 | | |
| Care-as-usual | ·· | ·· | ·· | 109 | 10·70 | 19·71 | 106 | 14·78 | 26·46 | 98 | 14·63 | 25·80 | 87 | 15·33 | 24·80 | | |
| Menopausal symptoms | | | | | | | | | | | | | | | | | |
| EORTC QLQ-CX24 (0-100) | | | | | | | | | | | | | | | | 4·27(8) | 0·83 |
| Intervention | ·· | ·· | ·· | 107 | 36·76 | 32·68 | 106 | 37·11 | 32·31 | 98 | 32·65 | 33·15 | 94 | 30·85 | 30·23 | | |
| Care-as-usual | ·· | ·· | ·· | 109 | 31·19 | 31·84 | 106 | 30·82 | 33·40 | 98 | 28·91 | 30·13 | 87 | 23·37 | 28·36 | | |
| Urological symptoms | | | | | | | | | | | | | | | | | |
| EORTC QLQ-CX24 (0-100) | | | | | | | | | | | | | | | | 3·32(8) | 0·91 |
| Intervention | ·· | ·· | ·· | 107 | 18·77 | 18·28 | 105 | 13·41 | 14·36 | 98 | 15·14 | 15·48 | 94 | 13·21 | 12·78 | | |
| Care-as-usual | ·· | ·· | ·· | 109 | 17·74 | 15·92 | 106 | 15·17 | 16·34 | 98 | 15·05 | 14·88 | 87 | 14·56 | 14·45 | | |
| Gastrointestinal symptoms | | | | | | | | | | | | | | | | | |
| EORTC QLQ-CX24 (0-100) | | | | | | | | | | | | | | | | 9·28(8) | 0·32 |
| Intervention | ·· | ·· | ·· | 107 | 10·67 | 10·60 | 106 | 12·81 | 13·67 | 98 | 12·50 | 13·18 | 94 | 12·59 | 13·54 | | |
| Care-as-usual | ·· | ·· | ·· | 109 | 13·91 | 14·35 | 106 | 13·13 | 14·36 | 98 | 14·20 | 14·86 | 87 | 11·59 | 12·48 | | |
| Fear of sexuality - FSQ fear of non-penetration sexual activity (0-20) | | | | | | | | | | | | | | | | 7·19(8) | 0·51 |
| Intervention | ·· | ·· | ·· | 107 | 4·33 | 4·10 | 106 | 3·56 | 3·94 | 98 | 3·58 | 4·39 | 94 | 4·11 | 4·73 | | |
| Care-as-usual | ·· | ·· | ·· | 109 | 3·83 | 4·10 | 106 | 3·40 | 3·83 | 98 | 3·30 | 3·80 | 87 | 3·32 | 4·30 | | |
| Fear of sexuality - FSQ fear of coitus/vaginal penetration (0-12) | | | | | | | | | | | | | | | | 2·41(8) | 0·97 |
| Intervention | ·· | ·· | ·· | 107 | 1·65 | 2·52 | 106 | 1·65 | 2·26 | 98 | 1·45 | 2·16 | 94 | 1·90 | 2·61 | | |
| Care-as-usual | ·· | ·· | ·· | 109 | 1·39 | 2·32 | 106 | 1·69 | 2·81 | 98 | 1·43 | 2·28 | 87 | 1·66 | 2·59 | | |
| Relationship dissatisfaction | | | | | | | | | | | | | | | | 4·79(8) | 0·78 |
| MMQ Marital scale | ·· | ·· | ·· | 90 | 10·06 | 11·53 | 89 | 9·97 | 11·57 | 83 | 10·29 | 11·74 | 78 | 11·45 | 13·30 | | |
| Intervention | ·· | ·· | ·· | 84 | 11·76 | 15·35 | 80 | 10·75 | 12·44 | 72 | 10·08 | 9·58 | 66 | 10·85 | 11·16 | | |
| Care-as-usual | | | | | | | | | | | | | | | | | |

**Supplementary appendix 3** *Continued*

| | Assessment | | | | | | | | | | | | | | | GLMM outcomes | |
|---|---|---|---|---|---|---|---|---|---|---|---|---|---|---|---|---|---|
| | T1 | | | T2 | | | T3 | | | T4 | | | T5 | | | | |
| Outcome measure (range score) | N | M/ % | SD | N | M/ % | SD | N | M/ % | SD | N | M/ % | SD | N | M/ % | SD | LRT(df) | P (overall model) |
| Psychological distress | | | | | | | | | | | | | | | | | |
| HADS Total (0-42) | | | | | | | | | | | | | | | | 5·87(8) | 0·66 |
| Intervention | ·· | ·· | ·· | 107 | 9·95 | 7·30 | 105 | 9·87 | 7·14 | 98 | 9·46 | 7·33 | 94 | 8·93 | 7·74 | | |
| Care-as-usual | ·· | ·· | ·· | 107 | 9·93 | 7·65 | 106 | 9·80 | 7·32 | 98 | 10·00 | 6·83 | 87 | 9·32 | 6·63 | | |
| Psychological distress | | | | | | | | | | | | | | | | | |
| HADS depression (0-21) | | | | | | | | | | | | | | | | 5·96(8) | 0·65 |
| Intervention | ·· | ·· | ·· | 107 | 4·22 | 3·68 | 105 | 4·13 | 3·89 | 98 | 3·87 | 3·62 | 94 | 3·47 | 3·79 | | |
| Care-as-usual | ·· | ·· | ·· | 107 | 4·36 | 4·03 | 106 | 4·01 | 3·86 | 98 | 4·21 | 3·64 | 87 | 3·41 | 3·36 | | |
| Psychological distress | | | | | | | | | | | | | | | | | |
| HADS anxiety (0-21) | | | | | | | | | | | | | | | | 7·48(8) | 0·49 |
| Intervention | ·· | ·· | ·· | 107 | 5·74 | 4·29 | 105 | 5·73 | 3·89 | 98 | 5·59 | 4·36 | 94 | 5·38 | 4·48 | | |
| Care-as-usual | ·· | ·· | ·· | 107 | 5·57 | 4·34 | 106 | 5·79 | 4·27 | 98 | 5·79 | 4·07 | 87 | 5·91 | 4·03 | | |
| Physical function | | | | | | | | | | | | | | | | | |
| EORTC QLQ-C30 (0-100) | | | | | | | | | | | | | | | | 8·28(8) | 0·41 |
| Intervention | ·· | ·· | ·· | 107 | 82·12 | 16·60 | 105 | 85·91 | 16·39 | 98 | 88·57 | 13·02 | 94 | 92·27 | 10·81 | | |
| Care-as-usual | ·· | ·· | ·· | 109 | 81·22 | 16·89 | 106 | 86·04 | 14·59 | 98 | 88·10 | 13·17 | 86 | 91·32 | 11·57 | | |
| Role function | | | | | | | | | | | | | | | | | |
| EORTC QLQ-C30 (0-100) | | | | | | | | | | | | | | | | 17·12(8) | 0·03* |
| Intervention | ·· | ·· | ·· | 107 | 66·04 | 30·45 | 106 | 71·23 | 27·65 | 98 | 72·79 | 29·05 | 94 | 80·50 | 24·15 | | |
| Care-as-usual | ·· | ·· | ·· | 109 | 66·67 | 25·36 | 106 | 69·81 | 25·73 | 98 | 75·17 | 23·96 | 87 | 82·95 | 23·29 | | |
| Emotional function | | | | | | | | | | | | | | | | | |
| EORTC QLQ-C30 (0-100) | | | | | | | | | | | | | | | | 9·16(8) | 0·33 |
| Intervention | ·· | ·· | ·· | 107 | 70·25 | 20·92 | 106 | 71·78 | 20·88 | 98 | 71·51 | 21·37 | 94 | 73·49 | 23·02 | | |
| Care-as-usual | ·· | ·· | ·· | 109 | 66·67 | 23·07 | 106 | 67·37 | 23·60 | 98 | 67·18 | 23·32 | 87 | 73·85 | 19·70 | | |
| Cognitive function | | | | | | | | | | | | | | | | | |
| EORTC QLQ-C30 (0-100) | | | | | | | | | | | | | | | | 11·47(8) | 0·18 |
| Intervention | ·· | ·· | ·· | 107 | 71·18 | 23·52 | 106 | 74·06 | 20·97 | 98 | 68·71 | 25·02 | 94 | 72·87 | 24·32 | | |
| Care-as-usual | ·· | ·· | ·· | 109 | 68·65 | 24·19 | 106 | 70·44 | 23·15 | 98 | 72·11 | 25·19 | 87 | 76·05 | 23·80 | | |
| Social function | | | | | | | | | | | | | | | | | |
| EORTC QLQ-C30 (0-100) | | | | | | | | | | | | | | | | 9·7(8) | 0·29 |
| Intervention | ·· | ·· | ·· | 107 | 67·76 | 27·31 | 106 | 73·74 | 25·09 | 98 | 76·53 | 24·27 | 94 | 83·51 | 19·48 | | |
| Care-as-usual | ·· | ·· | ·· | 109 | 68·20 | 26·11 | 106 | 73·11 | 24·08 | 98 | 77·38 | 20·20 | 87 | 82·38 | 20·70 | | |
| Fatigue | | | | | | | | | | | | | | | | | |
| EORTC QLQ-C30 (0-100) | | | | | | | | | | | | | | | | 22·63(8) | 0·004* |
| Intervention | ·· | ·· | ·· | 107 | 41·85 | 22·41 | 106 | 36·16 | 23·36 | 98 | 36·40 | 20·54 | 94 | 30·14 | 23·98 | | |
| Care-as-usual | ·· | ·· | ·· | 108 | 44·65 | 23·63 | 105 | 40·42 | 23·22 | 98 | 36·84 | 22·82 | 87 | 28·86 | 23·57 | | |

**Supplementary appendix 3** *Continued*

| | Assessment | | | | | | | | | | | | | | | GLMM outcomes | |
|---|---|---|---|---|---|---|---|---|---|---|---|---|---|---|---|---|---|
| | T1 | | | T2 | | | T3 | | | T4 | | | T5 | | | | |
| Outcome measure (range score) | N | M/ % | SD | N | M/ % | SD | N | M/ % | SD | N | M/ % | SD | N | M/ % | SD | LRT(df) | P (overall model) |
| Nausea/vomiting | | | | | | | | | | | | | | | | | |
| EORTC QLQ-C30 (0-100) | | | | | | | | | | | | | | | | 6·45(6) | 0·38 |
| Intervention | ·· | ·· | ·· | 107 | 5·61 | 10·97 | 106 | 7·55 | 16·92 | 97 | 7·05 | 14·60 | 94 | 6·74 | 13·23 | | |
| Care-as-usual | ·· | ·· | ·· | 109 | 8·26 | 12·75 | 106 | 5·35 | 14·47 | 98 | 5·61 | 10·42 | 87 | 4·02 | 8·79 | | |
| Pain | | | | | | | | | | | | | | | | | |
| EORTC QLQ-C30 (0-100) | | | | | | | | | | | | | | | | 6·34(8) | 0·61 |
| Intervention | ·· | ·· | ·· | 107 | 20·09 | 20·57 | 106 | 19·18 | 21·18 | 97 | 20·96 | 23·97 | 94 | 16·67 | 22·0 | | |
| Care-as-usual | ·· | ·· | ·· | 109 | 19·88 | 23·18 | 106 | 19·81 | 24·03 | 98 | 20·41 | 23·88 | 87 | 14·18 | 19·94 | | |
| Dyspnoea | | | | | | | | | | | | | | | | | |
| EORTC QLQ-C30 (0-100) | | | | | | | | | | | | | | | | 3·23(8) | 0·92 |
| Intervention | ·· | ·· | ·· | 107 | 17·45 | 23·50 | 106 | 11·95 | 17·33 | 98 | 9·18 | 18·40 | 94 | 10·28 | 20·75 | | |
| Care-as-usual | ·· | ·· | ·· | 109 | 16·82 | 21·58 | 106 | 14·47 | 18·98 | 98 | 11·22 | 21·38 | 87 | 7·66 | 15·83 | | |
| Sleep disturbance | | | | | | | | | | | | | | | | | |
| EORTC QLQ-C30 (0-100) | | | | | | | | | | | | | | | | 9·71(8) | 0·29 |
| Intervention | ·· | ·· | ·· | 107 | 36·14 | 29·0 | 106 | 32·39 | 31·02 | 98 | 31·63 | 29·65 | 94 | 30·14 | 31·71 | | |
| Care-as-usual | ·· | ·· | ·· | 109 | 30·89 | 29·64 | 106 | 28·62 | 30·32 | 98 | 29·59 | 26·17 | 87 | 27·20 | 27·15 | | |
| Appetite loss | | | | | | | | | | | | | | | | | |
| EORTC QLQ-C30 (0-100) | | | | | | | | | | | | | | | | 1·82(6) | 0·94 |
| Intervention | ·· | ·· | ·· | 107 | 9·66 | 15·87 | 106 | 10·06 | 19·61 | 98 | 8·84 | 18·88 | 94 | 6·74 | 16·0 | | |
| Care-as-usual | ·· | ·· | ·· | 109 | 13·76 | 22·32 | 106 | 7·86 | 17·55 | 98 | 7·48 | 16·25 | 87 | 6·13 | 15·69 | | |
| Constipation | | | | | | | | | | | | | | | | | |
| EORTC QLQ-C30 (0-100) | | | | | | | | | | | | | | | | 1·53(8) | 0·99 |
| Intervention | ·· | ·· | ·· | 107 | 10·28 | 18·55 | 106 | 10·06 | 18·50 | 97 | 12·72 | 21·75 | 94 | 10·28 | 18·31 | | |
| Care-as-usual | ·· | ·· | ·· | 109 | 10·40 | 22·54 | 106 | 6·60 | 15·55 | 98 | 11·57 | 24·92 | 87 | 7·28 | 18·63 | | |
| Diarrhoea | | | | | | | | | | | | | | | | | |
| EORTC QLQ-C30 (0-100) | | | | | | | | | | | | | | | | 3·51(8) | 0·90 |
| Intervention | ·· | ·· | ·· | 107 | 13·08 | 19·83 | 106 | 14·78 | 22·58 | 98 | 15·31 | 23·04 | 94 | 11·35 | 18·65 | | |
| Care-as-usual | ·· | ·· | ·· | 109 | 19·57 | 24·52 | 106 | 17·61 | 26·11 | 98 | 18·37 | 23·02 | 87 | 12·64 | 19·85 | | |
| Financial impact | | | | | | | | | | | | | | | | | |
| EORTC QLQ-C30 (0-100) | | | | | | | | | | | | | | | | 2·9(8) | 0·94 |
| Intervention | ·· | ·· | ·· | 107 | 12·46 | 23·58 | 106 | 13·21 | 23·76 | 98 | 11·91 | 20·45 | 94 | 11·35 | 21·06 | | |
| Care-as-usual | ·· | ·· | ·· | 109 | 15·60 | 25·49 | 106 | 11·01 | 22·41 | 98 | 10·20 | 20·54 | 87 | 14·18 | 25·74 | | |
| Global QoL | | | | | | | | | | | | | | | | | |
| EORTC QLQ-C30 (0-100) | | | | | | | | | | | | | | | | 7·2(8) | 0·51 |
| Intervention | ·· | ·· | ·· | 107 | 64·95 | 16·42 | 106 | 69·18 | 18·59 | 98 | 69·98 | 16·91 | 94 | 73·49 | 16·71 | | |
| Care-as-usual | ·· | ·· | ·· | 109 | 67·05 | 17·98 | 106 | 68·24 | 17·06 | 98 | 69·05 | 18·27 | 87 | 75·58 | 15·29 | | |

**Supplementary appendix 3** *Continued*

| Outcome measure (range score) | Assessment T1 N | T1 M/% | T1 SD | T2 N | T2 M/% | T2 SD | T3 N | T3 M/% | T3 SD | T4 N | T4 M/% | T4 SD | T5 N | T5 M/% | T5 SD | GLMM outcomes LRT(df) | P (overall model) |
|---|---|---|---|---|---|---|---|---|---|---|---|---|---|---|---|---|---|
| Gynaecological cancer treatment related stress | | | | | | | | | | | | | | | | | |
| IES Total (0-75) | | | | | | | | | | | | | | | | 6·03(8) | 0·64 |
| Intervention | ·· | ·· | ·· | 107 | 24·09 | 16·61 | 105 | 20·05 | 15·83 | 98 | 19·12 | 16·46 | 94 | 17·49 | 16·24 | | |
| Care-as-usual | ·· | ·· | ·· | 107 | 24·24 | 17·33 | 106 | 21·65 | 16·06 | 98 | 20·95 | 15·93 | 87 | 18·66 | 16·10 | | |
| **Physician-assessed** | | | | | | | | | | | | | | | | | |
| Stenosis (shortening/ tightening) CTCAE | | | | | | | | | | | | | | | | | |
| Intervention | | | | | | | | | | | | | | | | 8·83(5) | 0·12 |
| *Grade 0* | 96 | 90·6 | ·· | 75 | 83·3 | ·· | 62 | 70·5 | ·· | 57 | 64·0 | ·· | 56 | 63·6 | ·· | | |
| *Grade 1* | 8 | 7·5 | ·· | 11 | 12·2 | ·· | 20 | 22·7 | ·· | 26 | 29·2 | ·· | 21 | 23·9 | ·· | | |
| *Grade 2* | 1 | 0·9 | ·· | 4 | 4·4 | ·· | 5 | 5·7 | ·· | 2 | 2·2 | ·· | 10 | 11·4 | ·· | | |
| *Grade 3* | 1 | 0·9 | ·· | 0 | 0 | ·· | 1 | 1·1 | ·· | 4 | 4·5 | ·· | 1 | 1·1 | ·· | | |
| Care-as-usual | | | | | | | | | | | | | | | | | |
| *Grade 0* | 110 | 94·0 | ·· | 82 | 79·6 | ·· | 77 | 78·6 | ·· | 66 | 67·3 | ·· | 56 | 65·1 | ·· | | |
| *Grade 1* | 4 | 3·4 | ·· | 19 | 18·4 | ·· | 14 | 14·3 | ·· | 22 | 22·4 | ·· | 20 | 23·3 | ·· | | |
| *Grade 2* | 3 | 2·6 | ·· | 2 | 1·9 | ·· | 4 | 4·1 | ·· | 8 | 8·2 | ·· | 8 | 9·3 | ·· | | |
| *Grade 3* | 0 | 0 | ·· | 0 | 0 | ·· | 3 | 3·1 | ·· | 2 | 2·0 | ·· | 2 | 2·3 | ·· | | |
| Dryness CTCAE | | | | | | | | | | | | | | | | | |
| Intervention | | | | | | | | | | | | | | | | 4·64(5) | 0·46 |
| *Grade 0* | 99 | 96·1 | ·· | 77 | 85·6 | ·· | 57 | 65·5 | ·· | 59 | 68·6 | ·· | 60 | 70·6 | ·· | | |
| *Grade 1* | 2 | 1·9 | ·· | 12 | 13·3 | ·· | 20 | 23·0 | ·· | 17 | 19·8 | ·· | 18 | 21·2 | ·· | | |
| *Grade 2* | 1 | 1·0 | ·· | 1 | 1·1 | ·· | 10 | 11·5 | ·· | 8 | 9·3 | ·· | 6 | 7·1 | ·· | | |
| *Grade 3* | 1 | 1·0 | ·· | 0 | 0 | ·· | 0 | 0 | ·· | 2 | 2·3 | ·· | 1 | 1·2 | ·· | | |
| Care-as-usual | | | | | | | | | | | | | | | | | |
| *Grade 0* | 103 | 92·8 | ·· | 80 | 78·4 | ·· | 73 | 74·5 | ·· | 69 | 72·6 | ·· | 55 | 67·1 | ·· | | |
| *Grade 1* | 4 | 3·6 | ·· | 19 | 18·6 | ·· | 24 | 24·5 | ·· | 24 | 25·3 | ·· | 26 | 31·7 | ·· | | |
| *Grade 2* | 4 | 3·6 | ·· | 2 | 2·0 | ·· | 1 | 1·0 | ·· | 2 | 2·1 | ·· | 1 | 1·2 | ·· | | |
| *Grade 3* | 0 | 0 | ·· | 1 | 1·0 | ·· | 0 | 0 | ·· | 0 | 0 | ·· | 0 | 0 | ·· | | |
| Bleeding CTCAE | | | | | | | | | | | | | | | | | |
| Intervention | | | | | | | | | | | | | | | | 7·6(5) | 0·18 |
| *Grade 0* | 47 | 42·3 | ·· | 94 | 87·0 | ·· | 81 | 86·2 | ·· | 76 | 82·6 | ·· | 78 | 83·0 | ·· | | |
| *Grade 1* | 32 | 28·8 | ·· | 11 | 10·2 | ·· | 13 | 13·8 | ·· | 15 | 16·3 | ·· | 14 | 14·9 | ·· | | |
| *Grade 2* | 26 | 23·4 | ·· | 3 | 2·8 | ·· | 0 | 0 | ·· | 1 | 1·1 | ·· | 2 | 2·1 | ·· | | |
| *Grade 3* | 6 | 5·4 | ·· | 0 | 0 | ·· | 0 | 0 | ·· | 0 | 0 | ·· | 0 | 0 | ·· | | |
| Care-as-usual | | | | | | | | | | | | | | | | | |
| *Grade 0* | 51 | 43·6 | ·· | 102 | 91·1 | ·· | 88 | 81·5 | ·· | 78 | 77·2 | ·· | 72 | 80·0 | ·· | | |
| *Grade 1* | 32 | 27·4 | ·· | 10 | 8·9 | ·· | 19 | 17·6 | ·· | 22 | 21·8 | ·· | 17 | 18·9 | ·· | | |
| *Grade 2* | 31 | 26·5 | ·· | 0 | 0 | ·· | 1 | 0·9 | ·· | 1 | 1·0 | ·· | 1 | 1·1 | ·· | | |
| *Grade 3* | 3 | 2·6 | ·· | 0 | 0 | ·· | 0 | 0 | ·· | 0 | 0 | ·· | 0 | 0 | ·· | | |

**Supplementary appendix 3** *Continued*

| Outcome measure (range score) | T1 N | T1 M/% | T1 SD | T2 N | T2 M/% | T2 SD | T3 N | T3 M/% | T3 SD | T4 N | T4 M/% | T4 SD | T5 N | T5 M/% | T5 SD | GLMM outcomes LRT(df) | GLMM outcomes P (overall model) |
|---|---|---|---|---|---|---|---|---|---|---|---|---|---|---|---|---|---|
| Mucositis CTCAE | | | | | | | | | | | | | | | | | |
| Intervention | | | | | | | | | | | | | | | | 11·04(5) | 0·05* |
| *Grade 0* | 104 | 97·2 | ·· | 51 | 53·1 | ·· | 68 | 74·7 | ·· | 74 | 86·0 | ·· | 72 | 80·9 | ·· | | |
| *Grade 1* | 3 | 2·8 | ·· | 41 | 42·7 | ·· | 21 | 23·1 | ·· | 10 | 11·6 | ·· | 13 | 14·6 | ·· | | |
| *Grade 2* | 0 | 0 | ·· | 4 | 4·2 | ·· | 2 | 2·2 | ·· | 1 | 1·2 | ·· | 3 | 3·4 | ·· | | |
| *Grade 3* | 0 | 0 | ·· | 0 | 0 | ·· | 0 | 0 | ·· | 1 | 1·2 | ·· | 1 | 1·1 | ·· | | |
| *Grade 4* | 0 | 0 | ·· | 0 | 0 | ·· | 0 | 0 | ·· | 0 | 0 | ·· | 0 | 0 | ·· | | |
| Care-as-usual | | | | | | | | | | | | | | | | | |
| *Grade 0* | 111 | 95·7 | ·· | 54 | 50·9 | ·· | 68 | 71·6 | ·· | 81 | 84·4 | ·· | 70 | 82·4 | ·· | | |
| *Grade 1* | 4 | 3·4 | ·· | 46 | 43·4 | ·· | 21 | 22·1 | ·· | 12 | 12·5 | ·· | 13 | 15·3 | ·· | | |
| *Grade 2* | 1 | 0·9 | ·· | 5 | 4·7 | ·· | 2 | 2·1 | ·· | 2 | 2·1 | ·· | 2 | 2·4 | ·· | | |
| *Grade 3* | 0 | 0 | ·· | 1 | 0·9 | ·· | 3 | 3·2 | ·· | 1 | 1·0 | ·· | 0 | 0 | ·· | | |
| *Grade 4* | 0 | 0 | ·· | 0 | 0 | ·· | 1 | 1·1 | ·· | 0 | 0 | ·· | 0 | 0 | ·· | | |
| Discharge CTCAE | | | | | | | | | | | | | | | | | |
| Intervention | | | | | | | | | | | | | | | | 1·65(5) | 0·90 |
| *Grade 0* | 73 | 67·0 | ·· | 57 | 53·8 | ·· | 60 | 64·5 | ·· | 66 | 73·3 | ·· | 65 | 70·7 | ·· | | |
| *Grade 1* | 23 | 21·1 | ·· | 45 | 42·5 | ·· | 30 | 32·3 | ·· | 23 | 25·6 | ·· | 24 | 26·1 | ·· | | |
| *Grade 2* | 13 | 11·9 | ·· | 4 | 3·8 | ·· | 3 | 3·2 | ·· | 1 | 1·1 | ·· | 3 | 3·3 | ·· | | |
| *Grade 3* | 0 | 0 | ·· | 0 | 0 | ·· | 0 | 0 | ·· | 0 | 0 | ·· | 0 | 0 | ·· | | |
| Care-as-usual | | | | | | | | | | | | | | | | | |
| *Grade 0* | 70 | 61·4 | ·· | 56 | 50·5 | ·· | 66 | 63·5 | ·· | 69 | 70·4 | ·· | 62 | 68·9 | ·· | | |
| *Grade 1* | 33 | 28·9 | ·· | 43 | 38·7 | ·· | 29 | 27·9 | ·· | 20 | 20·4 | ·· | 27 | 30·0 | ·· | | |
| *Grade 2* | 11 | 9·6 | ·· | 12 | 10·8 | ·· | 9 | 8·7 | ·· | 9 | 9·2 | ·· | 1 | 1·1 | ·· | | |
| *Grade 3* | 0 | 0 | ·· | 0 | 0 | ·· | 0 | 0 | ·· | 0 | 0 | ·· | 0 | 0 | ·· | | |
| Fibrosis CTCAE | | | | | | | | | | | | | | | | | |
| Intervention | | | | | | | | | | | | | | | | 3·03(5) | 0·70 |
| *Grade 0* | 104 | 98·1 | ·· | 93 | 97·9 | ·· | 74 | 85·1 | ·· | 66 | 77·6 | ·· | 58 | 67·4 | ·· | | |
| *Grade 1* | 2 | 1·9 | ·· | 2 | 2·1 | ·· | 13 | 14·9 | ·· | 18 | 21·2 | ·· | 25 | 29·1 | ·· | | |
| *Grade 2* | 0 | 0 | ·· | 0 | 0 | ·· | 0 | 0 | ·· | 1 | 1·2 | ·· | 2 | 2·3 | ·· | | |
| *Grade 3* | 0 | 0 | ·· | 0 | 0 | ·· | 0 | 0 | ·· | 0 | 0 | ·· | 1 | 1·2 | ·· | | |
| Care-as-usual | | | | | | | | | | | | | | | | | |
| *Grade 0* | 111 | 96·5 | ·· | 88 | 88·9 | ·· | 79 | 85·9 | ·· | 77 | 80·2 | ·· | 60 | 72·3 | ·· | | |
| *Grade 1* | 3 | 2·6 | ·· | 11 | 11·1 | ·· | 13 | 14·1 | ·· | 15 | 15·6 | ·· | 22 | 26·5 | ·· | | |
| *Grade 2* | 1 | 0·9 | ·· | 0 | 0 | ·· | 0 | 0 | ·· | 3 | 3·1 | ·· | 1 | 1·2 | ·· | | |
| *Grade 3* | 0 | 0 | ·· | 0 | 0 | ·· | 0 | 0 | ·· | 1 | 1·0 | ·· | 0 | 0 | ·· | | |

**Supplementary appendix 3** *Continued*

| Outcome measure (range score) | T1 N | T1 M/% | T1 SD | T2 N | T2 M/% | T2 SD | T3 N | T3 M/% | T3 SD | T4 N | T4 M/% | T4 SD | T5 N | T5 M/% | T5 SD | GLMM outcomes LRT(df) | GLMM outcomes P (overall model) |
|---|---|---|---|---|---|---|---|---|---|---|---|---|---|---|---|---|---|
| Atrophy/telangiectasia CTCAE | | | | | | | | | | | | | | | | | |
| Intervention | | | | | | | | | | | | | | | | 6·29(4) | 0·18 |
| *Grade 0* | 103 | 97·2 | ·· | 81 | 87·1 | ·· | 65 | 73·9 | ·· | 51 | 63·0 | ·· | 42 | 49·4 | ·· | | |
| *Grade 1* | 2 | 1·9 | ·· | 12 | 12·9 | ·· | 23 | 26·1 | ·· | 29 | 35·8 | ·· | 35 | 41·2 | ·· | | |
| *Grade 2* | 1 | 0·9 | ·· | 0 | 0 | ·· | 0 | 0 | ·· | 1 | 1·2 | ·· | 6 | 7·1 | ·· | | |
| *Grade 3* | 0 | 0 | ·· | 0 | 0 | ·· | 0 | 0 | ·· | 0 | 0 | ·· | 1 | 1·2 | ·· | | |
| *Grade 4* | 0 | 0 | ·· | 0 | 0 | ·· | 0 | 0 | ·· | 0 | 0 | ·· | 1 | 1·2 | ·· | | |
| Care-as-usual | | | | | | | | | | | | | | | | | |
| *Grade 0* | 115 | 100·0 | ·· | 87 | 87·0 | ·· | 68 | 76·4 | ·· | 64 | 67·4 | ·· | 42 | 52·5 | ·· | | |
| *Grade 1* | 0 | 0 | ·· | 13 | 13·0 | ·· | 20 | 22·5 | ·· | 28 | 29·5 | ·· | 36 | 45·0 | ·· | | |
| *Grade 2* | 0 | 0 | ·· | 0 | 0 | ·· | 1 | 1·1 | ·· | 2 | 2·1 | ·· | 1 | 1·3 | ·· | | |
| *Grade 3* | 0 | 0 | ·· | 0 | 0 | ·· | 0 | 0 | ·· | 1 | 1·1 | ·· | 1 | 1·3 | ·· | | |
| *Grade 4* | 0 | 0 | ·· | 0 | 0 | ·· | 0 | 0 | ·· | 0 | 0 | ·· | 0 | 0 | ·· | | |
| Pain CTCAE | | | | | | | | | | | | | | | | | |
| Intervention | | | | | | | | | | | | | | | | 1·53(5) | 0·91 |
| *Grade 0* | 92 | 87·6 | ·· | 91 | 90·1 | ·· | 87 | 88·8 | ·· | 82 | 91·1 | ·· | 80 | 88·9 | ·· | | |
| *Grade 1* | 11 | 10·5 | ·· | 8 | 7·9 | ·· | 11 | 11·2 | ·· | 7 | 7·8 | ·· | 5 | 5·6 | ·· | | |
| *Grade 2* | 2 | 1·9 | ·· | 2 | 2·0 | ·· | 0 | 0 | ·· | 1 | 1·1 | ·· | 5 | 5·6 | ·· | | |
| *Grade 3* | 0 | 0 | ·· | 0 | 0 | ·· | 0 | 0 | ·· | 0 | 0 | ·· | 0 | 0 | ·· | | |
| Care-as-usual | | | | | | | | | | | | | | | | | |
| *Grade 0* | 107 | 92·2 | ·· | 90 | 80·4 | ·· | 94 | 87·9 | ·· | 87 | 88·8 | ·· | 80 | 90·9 | ·· | | |
| *Grade 1* | 7 | 6·0 | ·· | 20 | 17·9 | ·· | 9 | 8·4 | ·· | 8 | 8·2 | ·· | 8 | 9·1 | ·· | | |
| *Grade 2* | 2 | 1·7 | ·· | 2 | 1·8 | ·· | 3 | 2·8 | ·· | 2 | 2·0 | ·· | 0 | 0 | ·· | | |
| *Grade 3* | 0 | 0 | ·· | 0 | 0 | ·· | 1 | 0·9 | ·· | 1 | 1·0 | ·· | 0 | 0 | ·· | | |
| Dyspareunia CTCAE | | | | | | | | | | | | | | | | | |
| Intervention | | | | | | | | | | | | | | | | 1·37(5) | 0·93 |
| *Grade 0* | 74 | 91·4 | ·· | 45 | 80·4 | ·· | 45 | 62·5 | ·· | 50 | 61·7 | ·· | 54 | 64·3 | ·· | | |
| *Grade 1* | 3 | 3·7 | ·· | 9 | 16·1 | ·· | 21 | 29·2 | ·· | 24 | 29·6 | ·· | 24 | 28·6 | ·· | | |
| *Grade 2* | 2 | 2·5 | ·· | 2 | 3·6 | ·· | 5 | 6·9 | ·· | 5 | 6·2 | ·· | 4 | 4·8 | ·· | | |
| *Grade 3* | 2 | 2·5 | ·· | 0 | 0 | ·· | 1 | 1·4 | ·· | 2 | 2·5 | ·· | 2 | 2·4 | ·· | | |
| Care-as-usual | | | | | | | | | | | | | | | | | |
| *Grade 0* | 73 | 84·9 | ·· | 34 | 64·2 | ·· | 49 | 63·6 | ·· | 50 | 64·9 | ·· | 50 | 71·4 | ·· | | |
| *Grade 1* | 10 | 11·6 | ·· | 15 | 28·3 | ·· | 18 | 23·4 | ·· | 17 | 22·1 | ·· | 14 | 20·0 | ·· | | |
| *Grade 2* | 3 | 3·5 | ·· | 3 | 5·7 | ·· | 9 | 11·7 | ·· | 8 | 10·4 | ·· | 5 | 7·1 | ·· | | |
| *Grade 3* | 0 | 0 | ·· | 1 | 1·9 | ·· | 1 | 1·3 | ·· | 2 | 2·6 | ·· | 1 | 1·4 | ·· | | |

*Note*. CTCAE = Common Terminology Criteria for Adverse Events; df = degrees of freedom; EORTC QLQ-C30 = European Organization for Research and Treatment of Cancer Quality of Life Questionnaire-Core 30; EORTC QLQ-CX24 = European Organization for Research and Treatment of Cancer Quality of Life Questionnaire-Gynaecological Cancer Module; EORTC QLQ-EN24 = European Organization for Research and Treatment of Cancer Quality of Life Questionnaire-Endometrial Cancer Module; FSDS = Female Sexual Distress Scale; FSFI = Female Sexual Function Index; FSQ = Fear of Sexuality Questionnaire; GLMM = Generalized Linear Mixed effects Model; HADS = Hospital Anxiety and Depression Scale; IES = Impact of Event Scale; IQR = interquartile range; M = mean; Md = median; MMQ = Maudsley Marital Questionnaire; N = observed number of women at the specific timepoint; QoL = Quality of Life; SD = standard deviation.

*$p < 0·05$.

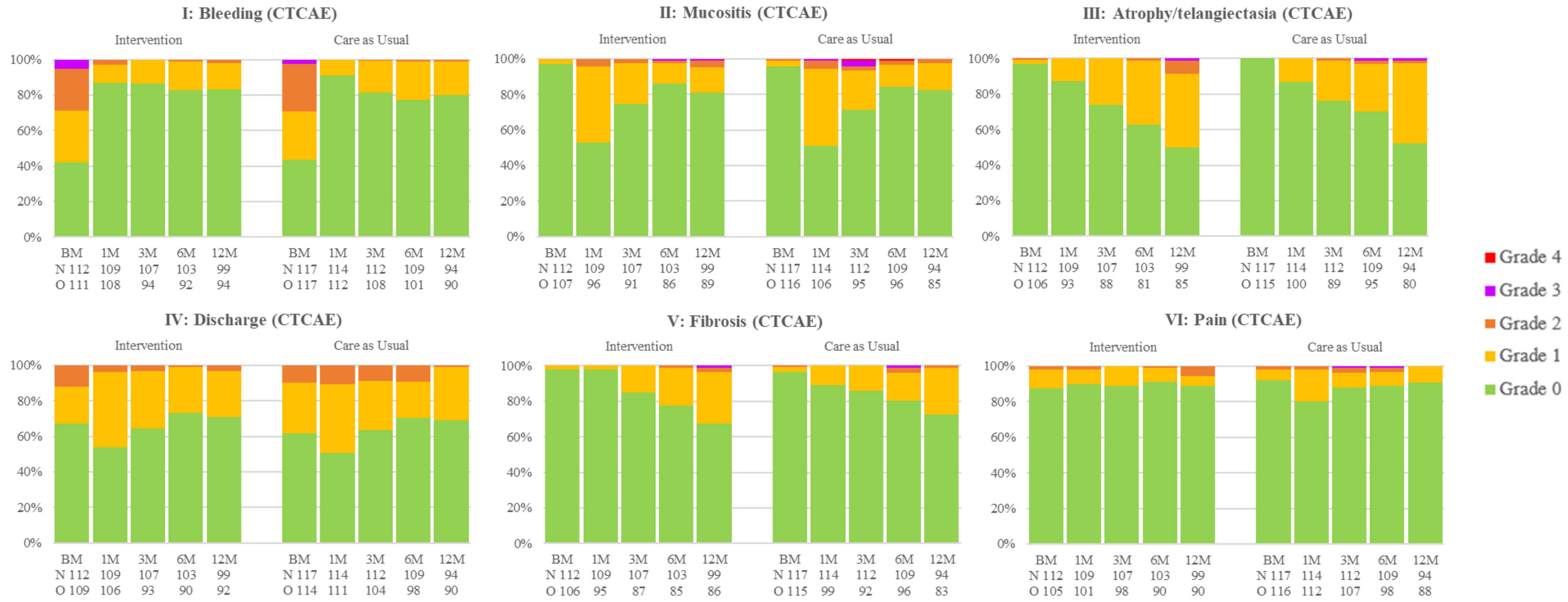


**Supplementary appendix 4** Physician-reported clinical measurements on single item level over time. The proportion of women is shown in percentages. BM = baseline measurement; CTCAE = Common Terminology Criteria for Adverse Events; M = months; N = number of women at risk at the specific timepoint; O = observed number of women at the specific timepoint.

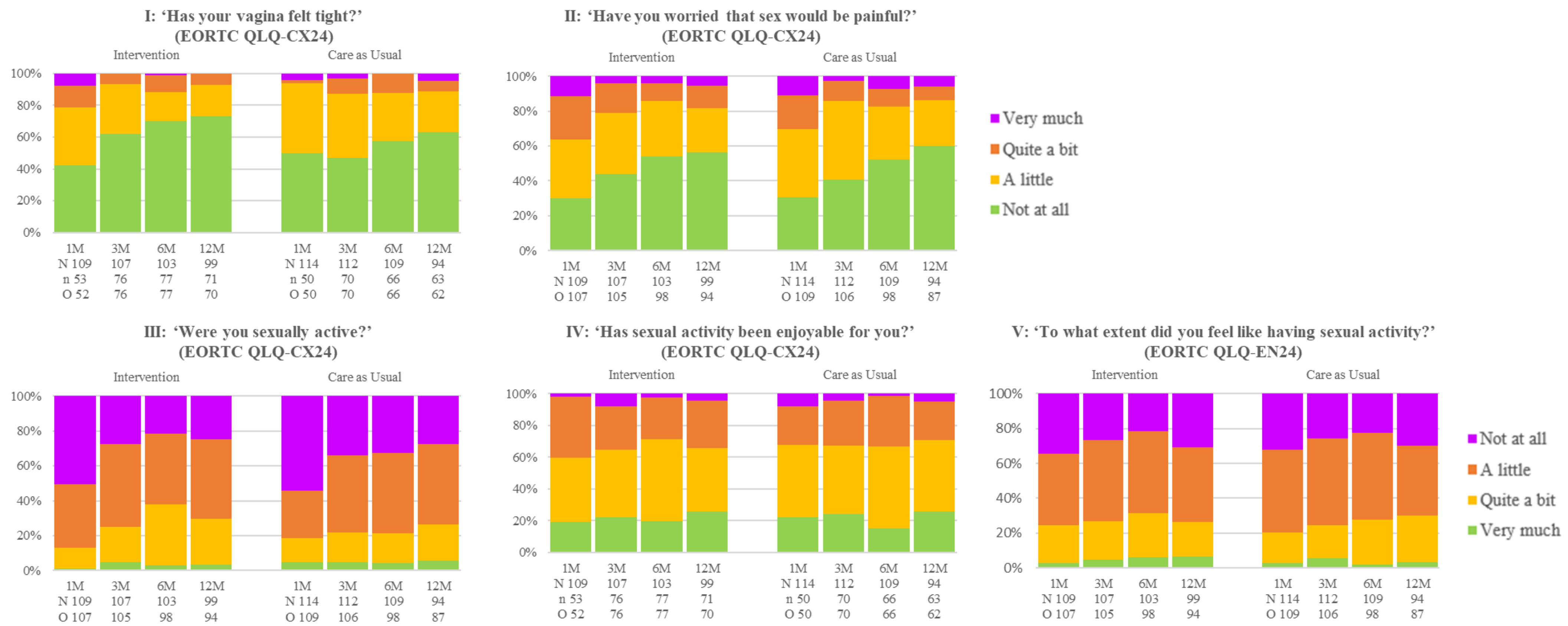


**Supplementary appendix 5** Patient-reported vaginal functioning problems on single item level over time. The proportion of women is shown in percentages. Figure I and IV only includes sexually active women. EORTC QLQ-CX24 = European Organization for Research and Treatment of Cancer Quality of Life Questionnaire-Gynaecological Cancer Module; EORTC QLQ-EN24 = European Organization for Research and Treatment of Cancer Quality of Life Questionnaire-Endometrial Cancer Module; M = months; N = number of women at risk at the specific timepoint; n = number of sexually active women at risk at the specific timepoint according to EORTC QLQ-CX24 item 19; O = observed number of women at the specific timepoint.

**Supplementary appendix 6 The prevalence of women scoring at a clinical level on sexual functioning, sexual distress, relationship dissatisfaction, and psychological distress**

| | N | n cut-off* | % cut-off* |
|---|---|---|---|
| **Primary Outcome** | | | |
| FSFItotal BM | | | |
| Intervention (total group) | 112 | 33 | 29·5 |
| Care-as-usual (total group) | 117 | 29 | 24·8 |
| | | | |
| Sexually active | 185 | 47 | 25·4 |
| Not sexually active | 42 | 14 | 33·3 |
| | | | |
| Sexual intercourse | 192 | 33 | 17·2 |
| No sexual intercourse | 35 | 28 | 80·0 |
| FSFItotal 1M | | | |
| Intervention (total group) | 108 | 80 | 74·1 |
| Care-as-usual (total group) | 110 | 82 | 74.5 |
| | | | |
| Sexually active | 128 | 79 | 61·7 |
| Not sexually active | 90 | 83 | 92·2 |
| | | | |
| Sexual intercourse | 84 | 38 | 45·2 |
| No sexual intercourse | 133 | 123 | 92·5 |
| FSFItotal 3M | | | |
| Intervention (total group) | 106 | 67 | 63·2 |
| Care-as-usual (total group) | 106 | 65 | 61.3 |
| | | | |
| Sexually active | 139 | 78 | 56·1 |
| Not sexually active | 73 | 54 | 74·0 |
| | | | |
| Sexual intercourse | 138 | 67 | 48·6 |
| No sexual intercourse | 74 | 65 | 87·8 |
| FSFItotal 6M | | | |
| Intervention (total group) | 98 | 49 | 50·0 |
| Care-as-usual (total group) | 98 | 54 | 55·1 |
| | | | |
| Sexually active | 128 | 59 | 46·1 |
| Not sexually active | 68 | 44 | 64·7 |
| | | | |
| Sexual intercourse | 134 | 48 | 35·8 |
| No sexual intercourse | 62 | 55 | 88·7 |
| FSFItotal 12M | | | |
| Intervention (total group) | 94 | 53 | 56·4 |
| Care-as-usual (total group) | 87 | 47 | 54·0 |
| | | | |
| Sexually active | 127 | 59 | 46·5 |
| Not sexually active | 54 | 41 | 75·9 |
| | | | |
| Sexual intercourse | 121 | 45 | 37·2 |
| No sexual intercourse | 60 | 55 | 91·7 |
| | | | |
| **Secondary Outcomes** | | | |
| FSDStotal BM | | | |
| Intervention | 112 | 29 | 25·9 |
| Care-as-usual | 117 | 30 | 25·6 |
| FSDStotal 1M | | | |
| Intervention | 107 | 54 | 50·5 |
| Care-as-usual | 110 | 49 | 44·5 |
| FSDStotal 3M | | | |
| Intervention | 106 | 49 | 46·2 |
| Care-as-usual | 106 | 45 | 42·5 |
| FSDStotal 6M | | | |
| Intervention | 98 | 43 | 43·9 |
| Care-as-usual | 98 | 46 | 46·9 |
| FSDStotal 12M | | | |
| Intervention | 94 | 42 | 44·7 |
| Care-as-usual | 87 | 40 | 46·0 |
| | | | |
| MMQtotal 1M | | | |
| Intervention | 90 | 13 | 14·4 |
| Care-as-usual | 84 | 16 | 19·0 |
| MMQtotal 3M | | | |
| Intervention | 89 | 16 | 18·0 |
| Care-as-usual | 80 | 15 | 18·8 |

**Supplementary appendix 6** *Continued*

| | | | |
|---|---|---|---|
| MMQtotal 6M | | | |
| Intervention | 83 | 17 | 20·5 |
| Care-as-usual | 72 | 11 | 15·3 |
| MMQtotal 12M | | | |
| Intervention | 78 | 15 | 19·2 |
| Care-as-usual | 66 | 13 | 19·7 |
| | | | |
| HADStotal 1M | | | |
| Intervention | 107 | 32 | 29·9 |
| Care-as-usual | 107 | 33 | 30·8 |
| HADStotal 3M | | | |
| Intervention | 105 | 33 | 31·4 |
| Care-as-usual | 106 | 27 | 25·5 |
| HADStotal 6M | | | |
| Intervention | 98 | 27 | 27·6 |
| Care-as-usual | 98 | 31 | 31·6 |
| HADStotal 12M | | | |
| Intervention | 94 | 21 | 22·3 |
| Care-as-usual | 87 | 21 | 24·1 |
| HADS depression 1M | | | |
| Intervention | 107 | 28 | 26·2 |
| Care-as-usual | 107 | 28 | 26·2 |
| HADS depression 3M | | | |
| Intervention | 105 | 25 | 23·8 |
| Care-as-usual | 106 | 20 | 18·9 |
| HADS depression 6M | | | |
| Intervention | 98 | 22 | 22·4 |
| Care-as-usual | 98 | 21 | 21·4 |
| HADS depression 12M | | | |
| Intervention | 94 | 21 | 22·3 |
| Care-as-usual | 87 | 16 | 18·4 |
| HADS anxiety 1M | | | |
| Intervention | 107 | 41 | 38·3 |
| Care-as-usual | 107 | 38 | 35·5 |
| HADS anxiety 3M | | | |
| Intervention | 105 | 43 | 41·0 |
| Care-as-usual | 106 | 41 | 38·7 |
| HADS anxiety 6M | | | |
| Intervention | 98 | 37 | 37·8 |
| Care-as-usual | 98 | 35 | 35·7 |
| HADS anxiety 12M | | | |
| Intervention | 94 | 31 | 33·0 |
| Care-as-usual | 87 | 36 | 41·4 |

*Note.* BM = baseline measurement; FSFI = Female Sexual Function Index; FSDS = Female Sexual Distress Scale; HADS = Hospital Anxiety and Depression Scale; M = months; MMQ = Maudsley Marital Questionnaire; N = observed number of women at the specific timepoint (for the MMQ, this number only includes women with a partner).
* = women that scored above/below the clinical cut-off point.
